# Development of a pKa predictor (pKaLearn) by leveraging teaching experience to improve machine learning

**DOI:** 10.1038/s42004-026-01983-y

**Published:** 2026-03-26

**Authors:** Jérôme Genzling, Ziling Luo, Benjamin Weiser, Nicolas Moitessier

**Affiliations:** Department of Chemistry, McGill University, Montreal, QC Canada

**Keywords:** Cheminformatics, Computational chemistry

## Abstract

Machine learning (ML) is gaining momentum in chemistry for the prediction of various molecular properties. However, these models are often trained on relatively scarce, sometimes low-quality data, resulting in what we describe as memorization (rather than learning) and poorly generalizable models. Aiming to revisit the way ML is practiced in chemistry, our strategy involves imparting chemistry knowledge to ML algorithms. Teachers teach chemistry with different levels of complexity in high school and graduate studies. This is due to fundamental principles being a prerequisite to understanding more advanced concepts. We posit that teaching fundamental principles to machines to predict properties, analogous to the way we teach students, will provide more accurate models. Thus, we propose to start with fundamental principles (e.g., electronegativity and inductive effect, conjugation, aromaticity) taught to students to allow them to predict properties (e.g., pKa) and provide these principles to machines to guide them to predict more advanced, yet related, properties. Based on this teaching-based approach, we developed pKaLearn, a pKa predictor that outperforms other state-of-the-art predictors. The ML models presented herein leverage the chemists’ knowledge and qualitative principles to quantify and predict chemical properties with high performance.

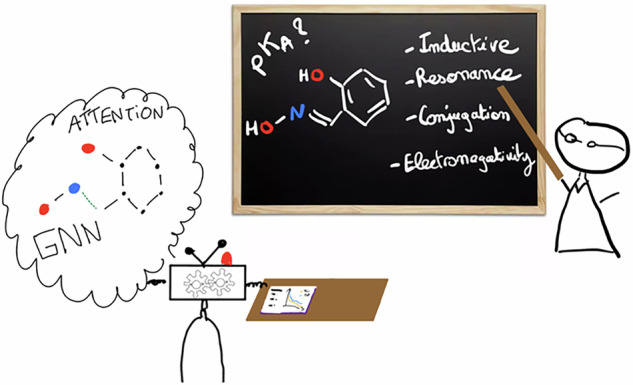

## Abstract (web summary)

Machine learning models for the prediction of molecular properties are often trained on relatively scarce data, resulting in memorization — rather than learning — and poor generalizability. Here, the authors develop a pKa predictor based on a teaching-based approach, demonstrating that the same fundamental principles that are taught to students — such as electronegativity, conjugation and aromaticity — can be used to train their machine learning model, which is shown to outperform state-of-the-art predictors.

## Introduction

### Teaching chemistry

When teaching in the classroom, a student will learn advanced chemistry more easily when previously taught fundamental principles. High school students learn about atoms, molecules, and their properties (e.g., electronegativity), while undergraduate students learn about nucleophilic substitution (e.g., polarized bonds due to electronegativity), stereochemistry, and frontier molecular orbitals (FMOs). Then, graduate students will, for example, learn the Felkin–Anh model predicting the stereochemical outcome of some reactions, a model rationalized by the FMO theory. These different levels can be put into practice in ML method development by teaching these principles to machines to optimize their learning of advanced principles.

### Artificial intelligence in chemistry

Artificial intelligence (AI) is revolutionizing the way chemistry research is carried out; in particular, the way drugs^1–5^ and materials^6–8^ are designed and discovered. In fact, in recent years, most large pharmaceutical companies have been adopting AI technologies, and small AI start-ups are burgeoning. However, we believe that successful AI technologies for chemistry would benefit from the synergy between computer science and experiments. Currently, the integration of these two fields is not necessarily happening since (1) models are often trained from structures and data with little domain expert guidance; (2) careful curation of the dataset by domain experts is required and selection of training and testing set is required to ensure reliable evaluation; (3) Experiments are required to generate large, uniform, and high-quality datasets. More specifically, while there are large datasets available for training popular methods (e.g., image recognition) where interpolation is often targeted, thousands of datapoints can hardly represent the chemical space estimated at 10^60^ ^9^, and extrapolation is required. These models, which are trained using relatively small datasets of molecular structures, or molecular fingerprints, are vulnerable to overtraining/memorization or inaccurate correlations, and physicochemical properties include subtle effects that require a sufficiently large amount of data to cover and quantify them.

Thus, developing an ML model often relies on the machine to discern the causes of the properties. In this case, either the computer is able to figure them all out, or it will memorize molecules (as seen in ML-based drug/protein binding scoring functions^10^). Another possible scenario is that the computer will come up with non-physical correlations and produce overtrained and poorly generalizable models

Our approach to computational chemistry methods is based on the encoding of well-established chemistry principles. For example, the Hoffman postulate was implemented into ACE to predict transition state structures^11^, while the impact of hyperconjugation on torsional energy was quantified in HTEQ^12^. Similarly, we wondered if we could act as teachers to improve ML models and apply this methodology to pKa prediction as a proof-of-concept. We should stress out that the main objective of this work is not to get the best pKa predictor possible, rather to validate our “teaching-like” approach to ML. Despite this original objective, our models (hereafter referred to as pKaLearn) were found to outperform several previously reported methods.

### Teaching and predicting pKa

While a student will more easily learn advanced chemistry when previously taught fundamental principles, these fundamental principles may be provided to computers (teaching) to optimize their learning of advanced principles, rather than providing the dataset and letting the computer self-teach. In addition, different levels of teaching may be considered depending on the complexity of the chemical concepts and their hierarchical relationships, as mentioned above. These different levels can be put into practice in ML method development.

**Fig. 1:**
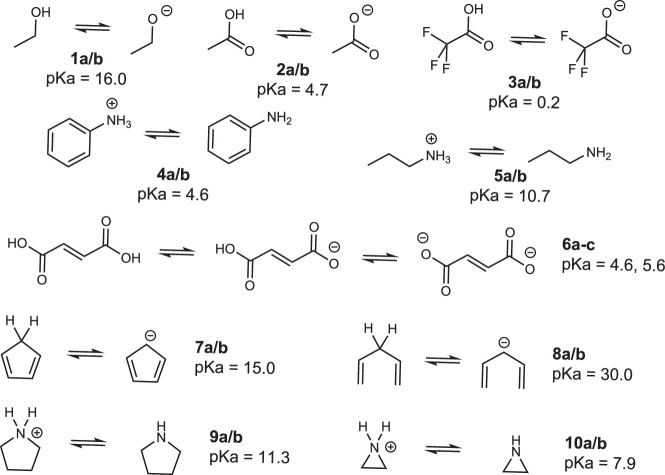
Teaching pKa trends. Structures and pKa values that illustrate the fundamental principles that affect pKa.

To illustrate our approach, we choose to predict the pKa of small molecules. To do so, fundamental principles taught in introductory organic chemistry courses were our starting points. When comparing the pK_a_ of simple organic molecules, teachers introduce resonance effects (**1** vs. **2**, Fig. 1) and inductive effects (**2** vs. **3**; **4** vs. **5**). Other effects known to impact pKa include the charge of the ionization center (**5a** vs. **5b**), the presence of other charges (**6a/b** vs. **6b/c**), the aromatization stabilization (**7** vs. **8**), and the presence/size of rings (**9** vs. **10**).

### Existing pKa predictors

Compared with conventional pK_a_ determination by experimental methods, computational models may provide alternative, universal, and affordable approaches. Quantum-mechanics (QM) based methods can predict pK_a_ yet with significant error (1.28 pK_a_ units as mean absolute error (MAE) for the method by Liao and Nicklaus)^13^. More recently, other models based on QM calculations have been developed to further improve the accuracy of the prediction, which reached an overall prediction error below 1.0 pK_a_ unit^14–21^. However, QM-based predictions still require considerable computational time and resources, which limit their applications to high-throughput molecular discoveries. Moreover, a failure to search for the accurate conformation may lead to substantial errors for conformationally sensitive structures; thus, more expensive molecular dynamics (MD) methods are commonly required in QM-based approaches^15,17,22–25^. Recently, methods such as the Conformer-Rotamer Ensemble Sampling Tool (CREST)^26^ have employed accurate semiempirical QM calculations, specifically using GFN2-xTB^27^. This method allows for faster yet accurate energy computations of the conformational search for various tautomers. On a single CPU core, this search requires seconds per molecule on average. Whereas this represents a significant improvement in speed due to recent computational advancements, the authors of the latest pKa predictor model QupKake, emphasize the need for a faster replacement for these semiempirical calculations^28^. Molecular mechanics-based MD methods have also been studied to further accelerate computational prediction of pK_a_ values^29,30^. In order to estimate the pK_a_ of proteins and large biological compounds, researchers have focused on developing empirical methods^31^ and methods based on computed dielectric constants^32–36^. These approaches could get most of the results within 1.0 pK_a_ unit error with relatively small data sets (300–500), although, once more, with a fairly low throughput.

With the emerging ML methods, pK_a_ predictors based on quantitative structure activity/property relationship (QSAR/QSPR) techniques have caught researchers’ attention^37–43^. The fundamental concept of QSPR models is to assume that structurally similar molecules tend to have similar properties. Thus, the mathematical relationship between pK_a_ values and descriptors/features of a set of training compounds can be constructed by linear regression^15,17,18,44^, support vector machine (SVM)^37,38,42,44^, random forest (RF)^38,42,44^, gradient boosting^42^, extreme gradient boosting (XGB)^37,38,42,44^, neural network (NN)^37,38,42,44^, and multiple other techniques^45^. The descriptors often represent structural and electronic information of the compounds (e.g., molecular weight, number of hydrogen bond donors, polar surface area). Alternatively, molecular fingerprints such as MACCS^46^, ECFP4^47^ (a specific instantiation of the Morgan algorithm^48^ with defined radius and bitstring length), and Estate^49^ fingerprints are used to encode molecule structures as binary strings, which, in turn, encode for the presence and absence of specific substructures, fragments, and chemical features. As an example, the holistic pK_a_ prediction studies^42^ based on the reliable and diverse iBond database (http://ibond.nankai.edu.cn)^50^ have reported that NN and XGB tend to perform better than other ML models (MAE = 0.87). A more recent study^44^ based on the S-pK_a_ dataset, which collected data from previous publications and other open-access pK_a_ databases, also proposed that the XGB model (MAE = 0.57 for acids, 0.55 for bases) outperformed SVM, RF, and NN models. Another study^38^ based on a different data set has shown that RF (MAE: 0.68, root mean square error (RMSE): 1.03, *R*^2^: 82%) could be a better candidate compared with XGB.

Overall, the reported ML methods predicting pKa are generally based on fingerprints or graphs encoding the structural and/or electronic information of the molecules^41,51–54^. However, while these models provide satisfactory accuracies (MAE below 1 pK_a_ unit), inductive or resonance effects are rarely mentioned. Instead, descriptors such as stereochemistry^44^ (no direct correlation with pKa) are sometimes used, or a method to automatically select the features may be opted for^40^. In addition, these models are trained on either one form (acid or base) or on acids and bases separately^44,52^. However, resonance and inductive stabilization may be observed in only one form. Finally, most published models in the literature do not discuss the similarities between their training and testing sets. This can lead to models memorizing the various chemical families they have seen, hence reducing the overall accuracy on compounds outside the training chemical space.

## Results and discussion

### Training and testing of pKaLearn, a pKa predictor

We first gauged the accuracy of our GAT model (see “Methods” section for details on this model). Through message passing, information from atoms up to seven bonds can be given to the ionization center when applying seven GAT layers. Alternatively, we could use a mask on the graph to conceal the atoms beyond the seven bond limits. One could also envision a combination of three GAT layers and a mask for any atoms more than four bonds away, as the atoms at the edge will encompass some information from the atoms in proximity to the edge (Section S-III). The influence of the local environment and the number of layers on the accuracy of the model was first tested. The training was carried out, providing the acidic/basic center associated with the pKa value.

**Fig. 2:**
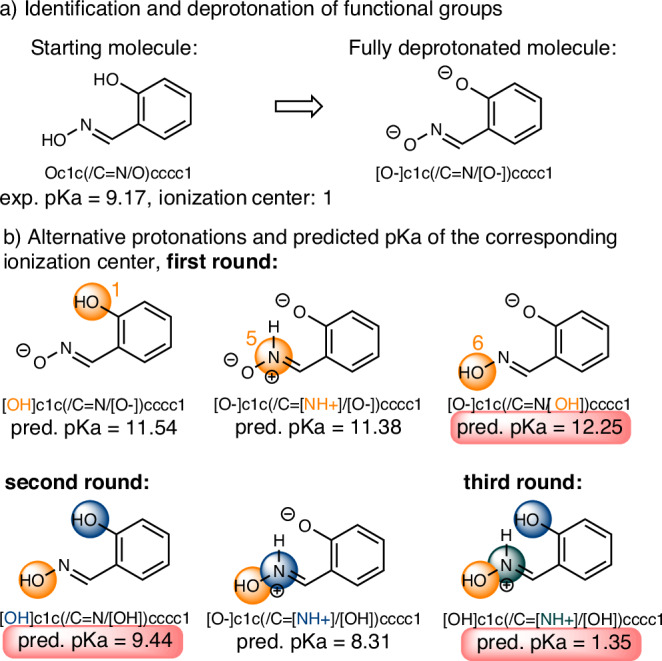
Prediction of multiple ionization centers. **a** Small molecules can have multiple possible protonation centers. The pKaLearn infer mode identifies all potential centers and generates the corresponding fully deprotonated state. **b** Starting from the deprotonated state, the model will iteratively evaluate the pKa for each intermediate protonation state to map the full ionization profile of the molecule.

However, while the model will be trained knowing the ionization center, predicting molecular pKa should not require this information. Our method was extended to evaluate various protonation states (microstates) as illustrated in Fig. 2. We coded the train of thought a chemist would go through to estimate pKa values on any molecule. The protocol starts with the identification of all the potential acidic sites. Then, these are deprotonated, and the model predicts the pKa of each site and identifies the most basic one. The latter is then protonated, and the pKa values of the other sites are recomputed, with the second most basic one identified and protonated. The process is iterated until all the basic sites are protonated and all the pKa values are computed. This method allows us not only to compute all these values but also to predict the most likely protonation state at a given pH (the pH can be given by the user), although we understand that multiple protonation states may co-exist.

**Fig. 3:**
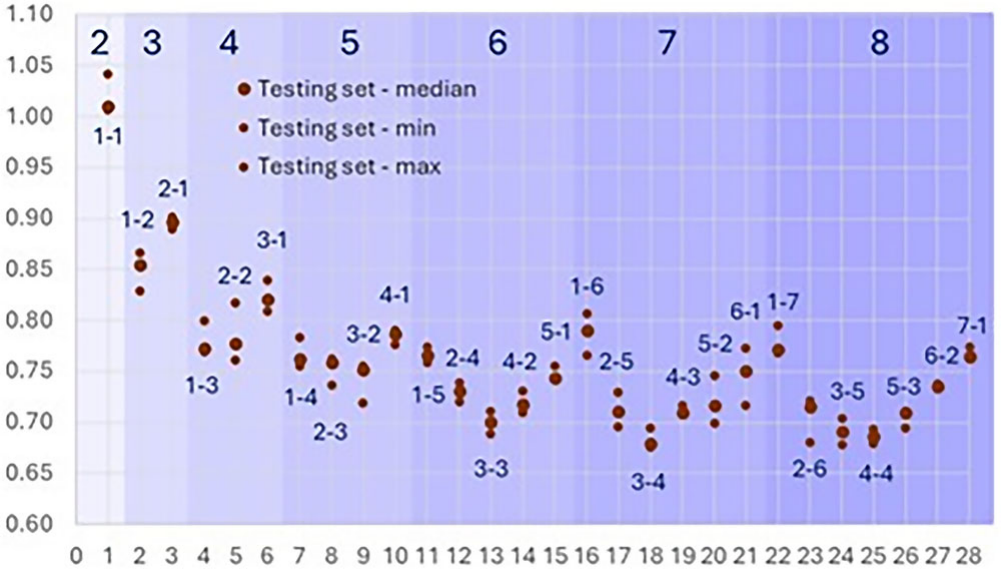
Effect of the number of layers. Evaluation (MAE) of models with different numbers of layers (1–6 denotes one GAT layer and a mask of size six, accounting for atoms seven bonds away, and is thus in the section labeled as “7” in the graph). The model was trained three times to evaluate the error related to the stochastic nature of the weight initialization.

From this first set of pKaLearn models, we first observed that the error of the models ranges between 0.70 and 0.80 pKa unit as long as the sum of the GAT layers and mask size is at least six (Fig. 3). A marked decrease in error is observed when going from a size of two (number of GAT layers + mask size) with a median MAE of 1.0 on the testing set to a size of three (0.88–0.91), 4 (0.77–0.84), and a minor decrease is then observed with more GAT layers and/or increased mask size. This error reached a minimum with a total size of seven or eight (0.67–0.74), in line with the trends observed experimentally (“Methods” section and Fig. S1). A size of two captures the nature of the ionization center (central atom and atoms directly bound). This layer depicts the necessary information to differentiate, for instance, a primary amine from a tertiary amine or an aniline. Additional long-range effects are captured with additional layers. These more subtle effects may at times be within the experimental error, leading to negligible improvement of the accuracy when stacking more GAT layers or increasing the mask size.

Unexpectedly, the relative number of layers and size of the mask had a substantial impact, with optimal accuracy observed when the number of GAT layers and mask size are balanced. As shown in Fig. 3, the models 3-3 (MAE = 0.69–0.71), 4-3 (0.71–0.72), 3-4 (0.68–0.69), 3-5 (0.68–0.70), 4-4 (0.68–0.69) and 5-3 (0.69–0.71) provided the most accurate predictions on the testing set. Overall, these models already demonstrated a significant improvement over baseline models shown in the “Methods” section.

### Proof-of-concept

**Fig. 4:**
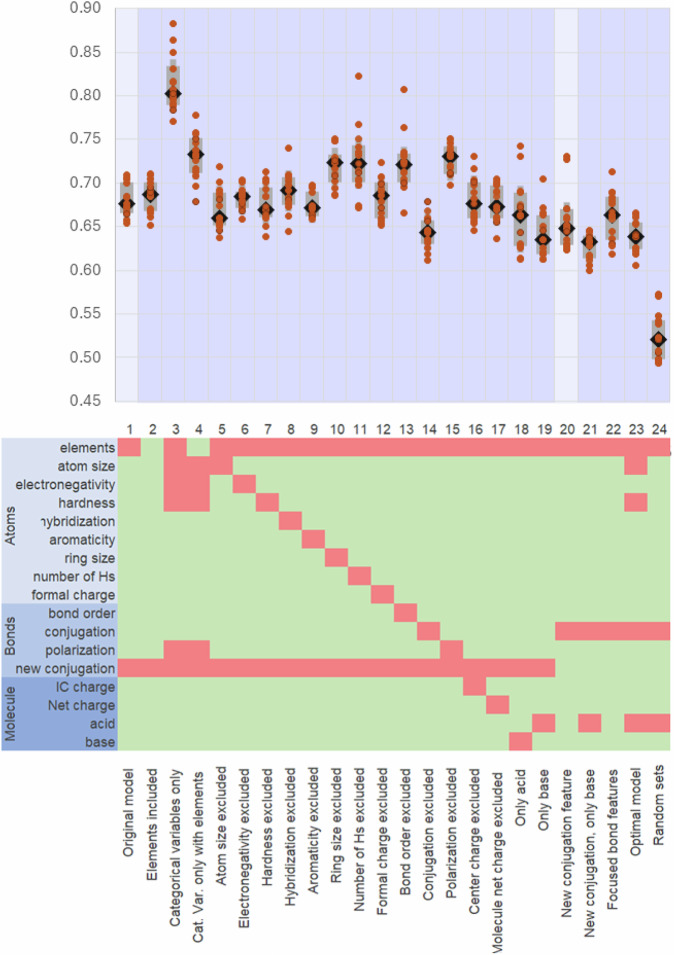
Impact of the features. Evaluation (MAE) of models with different features on the testing set. Top panel: accuracy of various models (MAE). The models 3-4, 3-5, 4-3, 4-4, and 5-3 were each trained three times, for a total of fifteen models developed for each condition. The dark diamonds refer to the median values with error bars in gray (20% to 80% percentiles). Full data is provided as supporting information. Models in column 1 (light blue background) are our reference models using the same conditions as in Fig. 3. Column 20 highlights the revised reference models after the conjugation feature was modified (see text). Cat. Var. stands for categorical variables. Bottom panel: features included (green)/excluded (red).

At this stage, a demonstration of the validity of our teaching-like strategy was needed. For this purpose, we trained the model (1) using only the categorical data variables, (2) providing the elements as done with many other previously reported models, and (3) removing some property-based features (e.g., hybridization, electronegativity, Fig. 4). These different conditions were applied to the models with the previously identified most appropriate number of GAT layers and mask size (3-4, 3-5, 4-3, 4-4 and 5-3) and the median accuracy was computed (Fig. 4).

Notably, adding the elements as features did not affect the accuracy of the model (entries 1 vs. 2; median MAE = 0.68 vs. 0.69), suggesting that the necessary atom-based information is captured by the variables included to account for inductive and resonance effects (e.g., electronegativity, bond polarization). In fact, when the non-categorical atom and bond features are removed and replaced by elements, the error increased (entries 1 vs 4, median MAE = 0.73). However, either elements or atomic properties are needed as shown with entry 3 (median MAE = 0.80) compared to entries 1 and 4. Overall, our approach provides models (MAE = 0.68 ± 0.02) which are significantly more accurate than the RF and XGB baseline models mentioned above (MAE > 0.9).

We then evaluated the impact of each feature on the model’s accuracy. As expected, removal of most of these features led to similar or slightly worse accuracy. Interestingly, removing atom size (entry 5, median MAE = 0.66) and hardness (entry 7, median MAE = 0.67) led to similar accuracies. As acidic atoms are mostly carbon, nitrogen, oxygen atoms, which are of similar size, and hardness is not directly linked to acidity, no drop in accuracy was expected while the slight improvement likely results from the simplification of the models. In contrast, removing ring size (entry 10, median MAE = 0.72) and the number of hydrogens (entry 11, median MAE = 0.72), two properties impacting pKa, led to significant drops.

More strikingly, removing conjugation led to a significant improvement (entry 1, median MAE = 0.68 vs. entry 14, median MAE = 0.64), while we believed this feature would be a key feature to model conjugation. A close look at the assignment of conjugated bonds in RDKit, the Python library used to compute conjugation, revealed that carboxylic acids and carboxylates are equally conjugated. Yet, the conjugation is more pronounced in carboxylates. To address this issue, we implemented our own conjugation feature and included it in our model (entry 20) as described in Supplementary Fig. 5. While the addition of the original conjugation feature decreased accuracy (from entry 14 to entry 1), introducing this revised conjugation definition does not (from entry 14 to entry 20). This new definition further increases the difference between our chemical principle-based models (entry 20, MAE = 0.65) and more categorical variable-based models (entry 4, MAE = 0.73) and traditional models (XGB, MAE = 0.92, “Methods” section) and further demonstrates the need for properly defined features rather than pre-established features. As a note, this RDKit function is used in many previously reported models (e.g., Chemprop^55^ and QupKake^28^), while we show here that it is not appropriate for pKa prediction. Similarly, removing bond order and bond polarization led to the largest losses (entry 1 vs. entries 13 and 15, median MAE = 0.72 and 0.73), indicating a more pronounced impact of bond features over atom features.

The molecular features (ionization formal charge and molecule net charge) added after the GAT layers were also removed one at a time (entries 16 and 17, median MAE = 0.68 and 0.67). The corresponding models showed accuracy similar to that of the original model. To test whether including features of both acids and bases was needed, we trained our model with either the acid or the base form (entries 19, MAE = 0.66, and 20, MAE = 0.64). These models appeared significantly more accurate than any models where both acids and bases were considered. However, the change in conjugation between acids and bases, resulting in enhanced resonance stabilization of the base as observed in carboxylic acids, is expected to be a major contributor, which requires both acids and bases. Once more, the conjugation definition in the RDKit library may be the cause of this unexpected behavior. The revised definition was used, the bases only were considered, and these derived models (entry 21) showed similar accuracy as the full models (entry 20). It demonstrated that the implemented conjugation definition is significantly more appropriate for pKa prediction and likely a better feature for resonance effect modeling.

Another possible reason for the improved accuracy observed when only acids or bases are used is the underrepresentation of acid/base pairs, where, for example, the aromaticity changes upon (de)protonation (e.g., 7a/b). Thus, including both acid and base features led to redundancy and/or unnecessary features and increased the size of the model (twice as many bond features). As a solution, some of these redundant features could be removed, and the model simplified and focused on the main changing features between acids and bases. A mode to focus on some of the features has been implemented. With this mode, the bond order in acids and bases is considered equivalent, so the bond order of acids is considered only. This focused mode reduces the number of bond features from 14 to 10. Unexpectedly, the accuracy is not improved (MAE = 0.66, entry 22).

### Model optimization and evaluation

From this first set of evaluations, we thought of generating an optimal pKaLearn model and testing it. First, removing atom size and hardness, which appeared non-important, did not impact the accuracy (model #23 in Fig. 4, MAE = 0.64).

As a test of the importance of proper evaluation of the “understanding” of the models, training and testing sets were generated by random splitting of the full dataset, as commonly done, and the model retrained (entry 24, median MAE = 0.52). The apparent increase in accuracy on the testing set, while no change was made to either the algorithm or parameters, emphasizes the need to test models using sets dissimilar to the training set to truly evaluate the accuracy of these models rather than evaluating what the models have memorized (not learned).

### Benchmark

As random splitting led to an MAE of ca. 0.52, our pKaLearn models seemed to compete with previously reported models, which used this training/testing set design. Fortunately, availability of the MolGpKa^52^ and Chemprop^55,56^ code allowed us to make a fair comparison under identical conditions. In contrast, we attempted to reconstruct the training pipeline for QupKake^28^, as no retraining script was provided. However, due to technical issues, we were unable to successfully replicate the retraining process in a timely fashion, and thus only MolGpKa and Chemprop were retrained on our datasets (Table 1, entry 2). Additionally, we compared the performance of the pKaLearn models and retrained MolGpKa and Chemprop on reported data. For this purpose, we used two previously reported testing sets (Novartis set and a set from Baltruschat et al. referred elsewhere to as a “literature” set)^52^. Unfortunately, we do not know the similarity between the training sets used to derive these other models shown in Table 2 and the Novartis and Baltruschat benchmark sets. In our original development discussed above, molecules from the Novartis and Baltruschat sets appeared in both the training and testing sets, and our sets had to be redesigned (Table 1, entries 3–6). In addition, as most of the existing models do not consider carbon atoms as ionization centers, they were removed from our sets. While this work was in progress, an updated version of Epik (Epik v. 7) came out, and its accuracy on a portion of the Novartis set is also provided in Table 2^54^.

**Table 1:** Performance (MAE) of our models

Entry	Training set	Testing set	Our pKaLearn models		w/Attentive FP		w/TransformerConv		Chemprop	MolGpKa
			w/out IC	w/ IC	w/out IC	w/ IC	w/out IC	w/ IC	w/out IC	w/out IC
1	Training set	Testing set	0.63 ± 0.02	0.60 ± 0.01	0.71 ± 0.02	0.68 ± 0.02	0.61 ± 0.01	0.62 ± 0.01	-	-
2	Training set no carbon^a^	Testing set no carbon^a^	0.59 ± 0.02	0.58 ± 0.01	0.65 ± 0.02	0.65 ± 0.01	0.58 ± 0.03	0.59 ± 0.02	0.62 ± 0.01	0.68 ± 0.02
3	FS^b^—(Novartis set^c^ and analogs)	Novartis set^c^	0.72 ± 0.03	0.76 ± 0.04	0.89 ± 0.05	1.05 ± 0.30	0.72 ± 0.03	0.76 ± 0.04	0.83 ± 0.01	0.89 ± 0.06
4	FS^b^—Novartis set^c^	Novartis set^c^	0.68 ± 0.03	0.71 ± 0.02	0.86 ± 0.06	1.01 ± 0.02	0.69 ± 0.04	0.74 ± 0.02	0.76 ± 0.03	0.88 ± 0.04
5	FS^b^—(Baltruschat set^c^ and analogs)	Baltruschat set^c^	0.40 ± 0.02	0.39 ± 0.02	0.42 ± 0.02	0.41 ± 0.16	0.40 ± 0.02	0.40 ± 0.02	0.43 ± 0.03	0.57 ± 0.06
6	FS^b^—Baltruschat set^c^	Baltruschat set^c^	0.34 ± 0.02	0.32 ± 0.02	0.36 ± 0.03	0.33 ± 0.03	0.35 ± 0.01	0.32 ± 0.01	0.36 ± 0.02	0.50 ± 0.04
7	Random training set^d^	Random testing set^d^	0.48 ± 0.01	0.49 ± 0.01	0.59 ± 0.01	0.55 ± 0.01	0.53 ± 0.01	0.51 ± 0.01	0.50 ± 0.01	0.62 ± 0.04

The performance is evaluated using the 4-4 models with GATv2Conv replaced by either Attentive FP or TransformerConv, each ran six times vs. Chemprop and MolGpKa. The error is the standard deviation over the six runs. Full data in Table S3.

^a^Training and testing sets used in other investigations above, excluding molecules with carbon atoms as ionization centers (IC).

^b^FS: our full set excluding molecules with carbon atoms as ionization centers (carbons are not considered as ionization centers by MolGpKa).

^c^Novartis: Novartis and Literature dataset as described in ref. ^38^.

^d^Random training and testing sets used above, excluding molecules with carbon atoms as ionization centers.

It is also worth noting that, during our curation of the benchmark sets reported by Baltruschat et al. (the “literature set”), we identified two molecules that were present in both the Novartis and the Literature sets. One of these molecules was associated with different (although similar; 4.3 vs. 4.5) experimental pKa values across the two sources, highlighting the error that may be incorporated when pKa values are determined using different techniques.

We also tested our pKaLearn models with the ionization center provided to the models (included in the input file) or not. In the latter case, our method will consider multiple ionization centers and ionization states (default mode) as discussed above (Fig. 2). Whereas this benchmarking considers data splitting, the various sets used, and their similarities, the training relied on one critical assumption: the ionization centers in our datasets are correctly identified and labeled. Indeed, while some molecules may have multiple functional groups that could be ionized around the same pH, there is no mention of any experimental validation of these ionization center assignments. To further benchmark these models, we could compare the predicted and experimentally proposed ionization site for each model. However, this approach might overlook false negatives in molecules with identical functional groups occurring twice (e.g., symmetric diols) or even molecules presenting ionization groups that are similarly acidic.

As can be seen in Table 1 (see Tables S3–S5 for complete data), the pKaLearn models trained on our training set with the molecules with carbon ionization centers removed (nitrogen, oxygen, and sulfur centers only) demonstrated an average error below 0.6 (entry 2, entry 1 includes carbon ionization centers) outperforming MolGpKa (median MAE = 0.68) and Chemprop (median MAE = 0.62). The testing on the Novartis set led to pKaLearn models with MAE around 0.70, whether the training set was designed to be different from this set (entry 3) or simply excluding all (entry 4) molecules from the Novartis set. Unexpectedly, when the ionization center is provided to the pKaLearn models and testing is carried out with the Novartis set, the error is larger than when the ionization is predicted by our models (Table 1, entry 3). A close look at the results revealed that the pKaLearn predicted ionization center is not always the one provided with the Novartis set, which was predicted (not experimentally determined) by the Marvin model. The discrepancies between Marvin and pKaLearn assignments are the major cause of the slight erosion of the accuracy when the Marvin-assigned ionization centers were provided to pKaLearn. In addition, this set is a small set of 280 compounds, meaning a small number of inaccurate predictions can lead to changes in the observed general performance. In parallel, testing on the set from Baltruschat et al. using a similar split of training and testing sets revealed pKaLearn models with an accuracy of 0.4 or below. We even reached MAE values of 0.32 ± 0.02 when the ionization center is given to the model. Similarly, when random training/testing sets were used, we once more observed an apparent reduction in error (entry 5), likely related to partial memorization and confirming the need for properly designed training and testing sets. We further compared the pKaLearn models to other reported methods (Table 2) and evaluated pKaLearn on the most recent blind challenges SAMPL8^57^ euroSAMPL1,^58^, comprising 23 and 35 compounds, respectively, with experimentally determined pKa values. Our models (entry 1, Table 3) achieved median MAE of 0.67 and 0.65 on these two sets. Based on the performance of models submitted to the original blind EuroSAMPL challenge, these results would place our approach among the more performing models, demonstrating strong generalization to unseen experimental data (Table 3).

Preliminary attempts to improve the pKaLearn models using other graph neural network (GNN) architectures (Attentive FP^59^ and a unified message passing model -UniMP- based on a graph transformer network - TransformerConv^60^) were not successful. While Attentive FP led to models consistently worse than our original pKaLearn models (ΔMAE between 0.1 and 0.4), the transformer-based architecture provided models with similar accuracy to our original GAT-based model. Our benchmark revealed that pKaLearn models outperform previously reported models, including MolGpKa, Ap-DNN, Marvin, Epik, and pkasolver, and have accuracies similar to QupKake. The performance reported for uni-pKa appears to be higher than with pKaLearn. However, the Novartis and SAMPL8^57^ datasets were part of the datasets used to develop this method^61^. In fact, we want to emphasize that the accuracies as presented in Tables 2 and 3 have to be taken with care as the training sets used to train these different models (1) vary and (2) may or may not include molecules from the Novartis, Baltruschat, and/or SAMPL8 test set. The only method that significantly outperforms pKaLearn is “SP1”. Unfortunately, there is no information on this anonymized method in the EuroSAMPL challenge report, and no other report on this method was found.

**Table 2:** Performance (MAE) of our models compared to other previously reported models

Entry	Model	Novartis Set		Baltruschat et al. set	
		MAE	RMSE	MAE	RMSE
1	pKaLearn models^a^	0.68 ± 0.03	0.86 ± 0.04	0.34 ± 0.02	0.52 ± 0.04
2	MolGpKa	0.81^52^	1.15^52^	0.49^78^	0.87^78^
3	AP-DNN	1.80^52^	2.45^52^	-	-
4	Marvin	0.80^52^–0.86^78^	1.17^78^	0.57^78^	0.87^78^
5	Epik (Epik v.7)	0.79^52^–0.83^78^ (0.43)^b^	1.16^78^	0.58^78^	0.92^78^
6	pkasolver	0.71^78^	0.93^78^	0.52^78^	0.82^78^
7	QupKake	0.58^28^	0.79^28^	0.40^28^	0.54^28^
8	Uni-pKa	0.62^61^	0.81^61^		

^a^Models developed above (Novartis set removed from the training set, same as Table 1, entry 4, and Baltruschat et al. test set removed from the training set, same as Table 1, entry 6), data provided in Table S4.

^b^MAE for Epik v.7, as some of the molecules of the Novartis set were in the training set, these were not considered in this testing. This MAE value was calculated based on data provided in ref. ^54^. As bases and acids are not equally represented in the Novartis set, the MAE values shown here are weighted averages of the reported MAE values on acids and bases.

**Table 3:** Performance (MAE) of our and other models on SAMPL8 and EuroSAMPL datasets (full data in Table S4)

Entry	Model	EuroSAMPL		SAMPL8	
		MAE	RMSE	MAE	RMSE
1	pKaLearn models^a^	0.65 ± 0.02	0.86 ± 0.04	0.67 ± 0.18	0.99 ± 0.27
2	SP1 (ML)	0.38	0.53		
3	r2SCAN-3c/DRACO + ML (QM + ML)	0.63	0.81		
4	CBio3Lab_p*K*_a_ (ML)	0.81	1.21		
5	BIOVIA COSMO-RS (QM)	0.70	1.39	0.95^b^	1.42^b^
6	QupKake (QM + ML)	0.78	1.67	0.62	1.04
7	H_2_O_DFT (QM)	1.41	1.76		
8	RIJCOSX-B3LYP-D3BJ(SMD)/cc-pV(T + d)Z (QM)	1.76	2.12		
9	IEFPCM_MST (QM)	2.00	2.57		
10	uESE (QM)	3.37	5.28		
11	EC-RISM (QM)	0.93	1.11		
12	Null hypothesis	2.14	2.44	2.38	4.08
13	Uni-pKa			0.63	0.88
14	Chemprop	0.66 ± 0.08	1.10 ± 0.07	0.75 ± 0.06	0.85 ± 0.06
15	MolGpKa	0.81 ± 0.10	0.92 ± 0.10	0.64 ± 0.08	1.02 ± 0.15

^a^Models developed above (same as Table 1, entry 2).

^b^Calculated from data provided as supporting information in ref. ^79^ Data for EuroSAMPL from ref. ^58^, data for SAMPL8 from refs. ^28,61,79^.

**Fig. 5:**
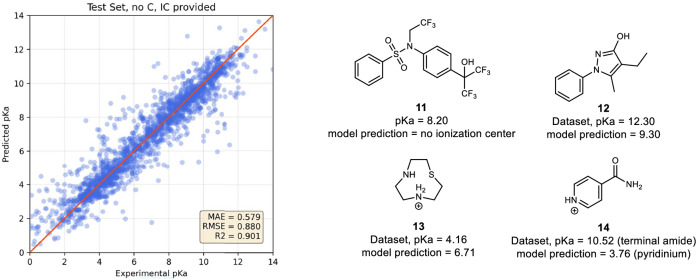
pKaLearn accuracy. Left panel: pKa predictions of our best performing model versus the labeled pKa values of our testing set with no carbons (Table 1, entry 2, 4-4 training #3). The *y* = *x* line (perfect prediction) is shown in red. Right panel: performances (MAE) of different models on our test sets. These models have been trained on various sets as described when originally developed.

Visualizing predicted pKa values versus experimental pKa values allowed us to better evaluate the accuracy of the different models. At this stage, identifying outliers is essential as they can reveal systematic errors and biases in the models. As shown in Fig. 5 and Supplementary Figs. 6–8, some molecules in the test set have large prediction errors (up to 10 pKa units) while some are predicted to have no ionization center. These errors might not be only due to the prediction but can also come from choices made in our encoding of the different chemical functional groups recognized by our model. For instance, we did not encode the recognition of simple alcohols bound to sp3 carbons as potential ionization centers, as most of these groups would present a pKa value over 14. Thus, the acidic alcohol in molecule **11** (activated by 2 trifluoromethyl groups) is not predicted, but when the ionization center is provided, a pKa of 8.82 (error: 0.62) is predicted. Tautomers may also be a problem when predicting pKa values. For example, molecule **12** has two tautomers while the pKa is predicted for only one of them, regardless of the relative stability of these tautomers and their conjugate bases. Internal hydrogen bonds, such as in acid **13**, are also disregarded in our model. Finally, the quality of the dataset, despite our major effort to curate it, remains a challenge. For example, the pKa of isonicotinamde **14** is experimentally determined at 10.5 in our set, while unfunctionalized pyridinium has a pKa of 5.2. The presence of the electron-withdrawing group in the para position is expected to further lower the pKa. Thus, the predicted value (3.76) is not necessarily wrong, while the experimental value is questionable. A search for the pKa value of isonicotinamide in other databases confirmed our view. Values ranging from 10.5 to 11.5^62,63^ likely corresponding to the terminal amide protons, and a value of 3.61^64^, likely corresponding to the pyridinium, have been reported. In this case, the ionization center that is predicted is simply not the one measured. In our ionization center identification, amides are considered acidic only if activated (not terminal amides). Thus, this potential center is not considered at all. Considering the total number of data points, the number of outliers identified from the plots is relatively small. As a note, a number of outliers are analogs of the same chemical families (e.g., thiazole, imidazole, Supplementary Fig. 11).

To evaluate the strengths and weaknesses of our model, we analyzed the combined results from all test sets (Table 1, entries 2, 4 and 6; Table 3, entry 1) to investigate prediction errors relative to the chemical environment of the protonation site. We averaged the prediction errors for duplicate molecules across multiple runs and classified each pKa site into one of the 18 functional groups we identified based on the GNN’s internal sub-graph logic. The resulting distribution of absolute error |∆pKa| is presented in Supplementary Fig. 12. As shown, the mean average error per chemical group remains consistently low, ranging between 0.15 and 0.45 units for all classes (except for a single Selenium containing molecule). Only 34 molecules exhibited an error exceeding 2 pKa units, demonstrating that our model generalizes effectively across the entire encountered chemical space. An interactive visualization of this chemical space, including group-specific filtering, is available on our GitHub repository (Chemical_space_test_sets.html).

### Randomized graphs and SMILES representation

Through the course of our work, we found that different SMILES for the same molecules (i.e., same graph but represented by matrices in different orders) sometimes led to different predicted pKa values. To test whether our approach using an augmented data set using randomized graphs is addressing this issue, each SMILES from the test sets was randomized five times, and these sets were used to test our models (Tables S5 and S6). In addition, we trained our models using a single graph (not randomized) directly derived from the SMILES provided in the training sets. The collected data (Tables S5 and S6) revealed that the use of randomized graphs and/or randomized SMILES did not significantly improve or deteriorate the model’s performance, whether pKaLearn models, Chemprop, or MolGpKa. With pKaLearn models, while the performance appears slightly worse with the Novartis set and slightly better with Baltruschat’s set, the differences are within the standard deviation of the methods.

### Effect of tautomers

Tautomers can significantly influence how models perceive molecular structures and therefore alter pKa predictions. As previously discussed, capturing the correct tautomeric form is particularly important for models aiming to predict microscopic pKa values, and several recent approaches have attempted to address this challenge^65^.

**Fig. 6:**
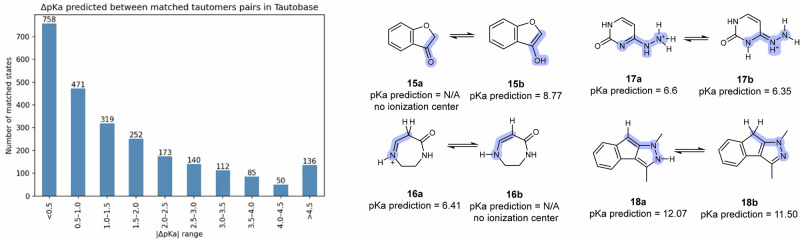
pKa of Tautomers. Left panel: Distribution of absolute pKa prediction differences (ΔpKa) across tautomeric state pairs recognized by our model. Right panel: Predictions of our models on various pairs of the Tautobase set.

To investigate the impact of tautomers on our model’s predictions, we used the Tautobase dataset^66^ that gathers 1680 pairs of tautomers. After removing pairs with no ionization centers and entries where no protonation state could be assigned, we obtained a final set of 1665 pairs in which at least one tautomeric form was predicted as acidic/basic by our model. Among these, 1460 pairs contained predictions for both tautomers (with some species having more than one protonation site). We matched protonation states based on overall molecular charge and computed the pKa differences between corresponding tautomeric states (Fig. 6).

In a small number of cases (205 pairs of molecules in the full Tautobase, 12% in total), errors arise from our model not recognizing certain functional groups as ionization centers (e.g., the acidic carbon α to a carbonyl in molecule **15a**, or the enamine nitrogen in molecule **16b**). However, for other tautomeric pairs, the model generally captures the expected behavior, with half of the pKa predictions of tautomers falling within 1 pKa unit of each other (49% of the 2496 predicted ionized pairs). These results show that while the model performs adequately on many tautomeric pairs, further work is needed to improve consistency across challenging cases, whether a site is not identified or when tautomerism can cause large discrepancies in pKa prediction between tautomers. Ongoing work in our group is focused on improving tautomer recognition and microscopic pKa consistency across tautomeric states.

## Conclusions

Through this work, we are proposing a knowledge-based design and evaluation of ML models for molecular property prediction. We selected descriptors (either atom or bond properties) directly from well-established chemical principles known to influence acidity and basicity. By guiding the model with such chemically meaningful features, we aimed to improve interpretability and generalizability, while demonstrating that this teaching-inspired strategy can reach state-of-the-art performance. More specifically, we propose to use chemical principles taught in class as starting points for designing the model rather than letting the machine figure out the necessary features with little guidance, an approach closer to self-teaching than to teaching. As a proof-of-principle, we applied this strategy to the prediction of pKa values. As the acidity/basicity of molecules is influenced by resonance, inductive, and other effects, we selected descriptors directly related to these properties. The selection and curation of these descriptors were thoroughly tested, resulting in the simplest yet complete set of chemical descriptors needed for our training. Gratifyingly, this approach led to a model that is more precise than a fingerprint-based model with minimal physical meaning, a GAT-based model, but only using categorical variables and other reported models. Moreover, we found that the model’s inference mode, which identifies potential ionization centers directly from the molecular structure, coupled with its test mode for probing specific hypotheses, offers a versatile dual-tool framework for users. While this work was primarily aimed at testing this concept of teaching machines, we were happy to see that this approach produced one of the most predictive pKa prediction models on a regular benchmark set.

## Methods

### Measuring effects

**Fig. 7:**
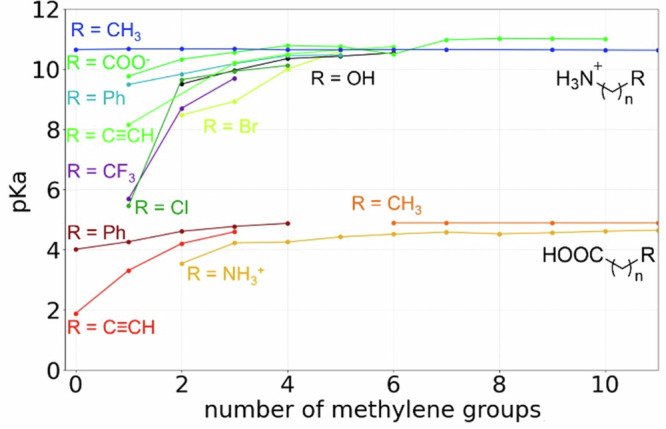
Inductive effect and pKa. Inductive effect as a function of the distance between the EWG/EDG and the ionization center.

To develop a model that best represents these resonances, inductive, and other effects mentioned above, we first investigated how far the resonance and inductive effects could travel. As can be seen in Fig. 7 and Supplementary Fig. 1, electron-withdrawing and donating groups (EWG, EDG) in aliphatic molecules modulate pKa even if it is as far as seven bonds away. Thus, our method should include the effect of atoms at least up to seven bonds away from the ionization center, but could ignore atoms further away without an expected loss of accuracy.

### Evaluating models

When evaluating students, we often aim to evaluate their understanding of the method, not necessarily their ability to memorize answers. Commonly, the dataset used for the development of ML models is randomly split into the training (examples in class) and testing sets (questions in exams)^44,54^. When these sets are similar, students/models memorizing answers may do just as well as students/models understanding the class material. To ensure understanding will be evaluated (and the model will be generalizable), we assembled and carefully curated a dataset comprising approximately 13,000 small molecule pKa values. To reduce the overlap between the training and the testing sets, we used a clustering-based split methodology, a strategy we have recently reported in the context of ML-augmented docking^67^. This strategy has specifically been designed to generate testing and training that are dissimilar. Benchmarking has been conducted using published and commonly used test sets from the literature. All the specific details can be found in the Supplementary Material (Section S-II).

### Baseline model

**Fig. 8:**
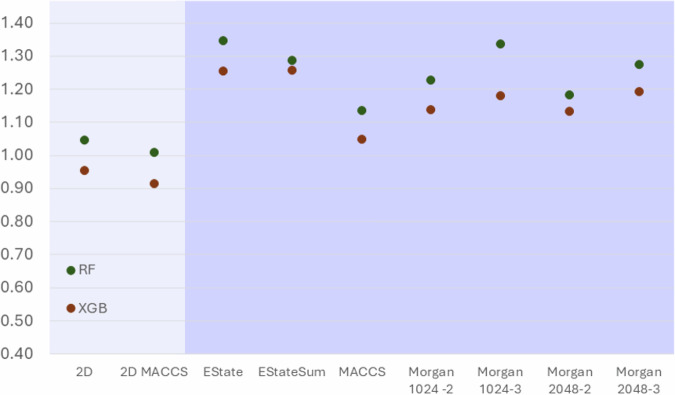
Reference models. Evaluation (MAE) of baseline RF (green) and XGB (orange) models with various featurizations. Morgan 1024-2 indicates the use of the Morgan fingerprint with a radius of 2 and 1024 bits. The light background highlights the most accurate models.

Prior to developing a complex model, we thought of establishing a baseline model. To proceed, we used RF and XGB algorithms, which are broadly used to predict pKa, and trained them using our own dataset and a variety of molecular descriptors or fingerprints (2D molecular descriptors, Estate, EstateSum, MACCS, 2D and MACCS combined, and Morgan fingerprints, Fig. 8). The optimal models (2D and 2D MACCS) achieved an MAE on our testing set of 0.96 (RF) and 0.92 (XGB). Moreover, these models were trained only to predict the global macro-pKa of a molecule based on its encoding through various descriptors.

### Graph neural network

Choosing an algorithm (equivalent to learning method/styles in classes) is the next step. The concept of GNN coincides with the basic components of molecules made of atoms (nodes) connected by chemical bonds (edges), making GNNs popular candidates to predict properties from graphs representing molecules. GNN’s have been used in recent reports to predict pKa’s as their main task^38,52,68^. The idea of getting knowledge through the structure of the graphic nature of molecules was first tested to get faster quantum chemistry energies from a molecule, using a Message-Passing Neural Network (MPNN)^69^. In addition, transfer learning strategies based on GNNs have also been recently proposed^70^. This study highlights that GNNs can effectively leverage knowledge from related properties to improve predictions on smaller pKa datasets, a challenge also addressed in our work but from a chemical-knowledge–driven perspective. For this work, we opted for a GNN with a multi-head attention mechanism (GAT) and more specifically the improved variant GATv2 reported by Brody et al.^71^. With this architecture, the spatial relationship between atoms is accounted for, thus allowing considerations of inductive and resonance effects. In other words, this GAT model can be “taught” the principles of inductive and resonance effects (Section S-III). To verify whether the GAT model was the best choice for our study, we developed and tested other models, as discussed below.

The next step was to select the features (i.e., properties) the GNN will learn from. Following the principles discussed above, the atoms will be represented by the node feature matrix, which contains properties of each atom, chosen to capture inductive resonance, and other effects known to impact pKa values.

- Selected atomic properties provide a first level of teaching: atomic electronegativity, hardness (inductive effects), as well as atomic diameter,
- Formal charge is also associated with acidity (**5a** vs. **5b**, Fig. 1),
- Hybridization affects pKa (e.g., terminal alkynes are more acidic than alkenes, in turn, more acidic than alkanes; iminium is more acidic than ammonium). (R-CH=NH vs. R-CH_2_-NH_2_),
- Ring size influences pKa (**9a/b** vs. **10a/b**, Fig. 1),
- Number of hydrogens; the local environment also affects pKa.
- The molecular net charge and the ionization center’s formal charge are two factors modulating pKa. Two bits are added to the embedding (after graph layers), which is then processed through two fully connected layers.

In contrast to most GNN’s using primarily categorical data variables (represented by a 1 or 0), our model is also trained on interval variables (e.g., electronegativity) normalized in the range [0, 1]. To guide the model training, the center of ionization is always provided as the first atom.

The edge feature matrix is used to represent the bond properties, including not only the bond type (single, aromatic, double, and triple bond) but also conjugation and bond polarization. Polarization is computed as the difference in electronegativity between the atoms making up each bond. To ensure the impact of this polarization on the ionization center is considered, the polarization should be directed (donating or withdrawing). To account for this direction, the polarization is computed as the difference between the atom further away and the one closer to the ionization center. These features are expected to model resonance and inductive effects. To test this hypothesis in detail, we systematically evaluated the impact of each feature on model performance and compared our architecture to a widely used “standard” GNN baseline, Chemprop^55,56^. The objective was to determine whether our chemistry-driven feature selection strategy provides measurable benefits over a conventional MPNN approach. In particular, our model incorporates physically motivated atomic descriptors—electronegativity, hardness (to capture inductive effects), and atomic diameter—which are absent in Chemprop. On the contrary, we decided to exclude chirality or atomic mass features, as we hypothesized these factors have minimal influence on pKa prediction.

### Technical details

All the features described above were computed using the RDKit package^72^ version 2022.3.5 (and 2025.3.6, versions varied on clusters from Digital Research Alliance of Canada and local workstations), apart from one modification we included regarding conjugation (Section S-IV). These features are then combined in a molecular graph representation of the molecule, encoded using the PyTorch Geometric package (version 2.6.1)^73^. We then trained our GNN models using Python version 3.11, Pytorch^74^ versions 2.4.1 (and 2.6.0, 2.8.0), and scikit-learn^75^ version 1.5.2 (and 1.6.1). More details can be found in our GitHub repository. The various models were trained on Digital Research Alliance of Canada’s supercomputers (mainly Narval and Graham clusters), using A100 GPU cards. Hyperparameters (learning rate, hidden layer dimension, dropout, number of GNN layers, and batch size) were optimized using a grid search on the training set. Because the dataset splitting already enforced low similarity between training and test molecules (Tanimoto coefficient ≤0.65), we did not employ additional cross-validation or nested cross-validation. Instead, each experiment was repeated three times with different random seeds to account for stochastic variation in initialization and optimization, and the median test set performance is reported.

### Data augmentation

In practice, a molecule may be represented by several graphs differing in the order of the atoms. Due to the variability of this SMILES representation, it has been shown that including this behavior may improve the quality of SMILES-based models^76^. As demonstrated by Mizuno and co-workers, canonical SMILES vary across toolkits and may not be the solution^77^. To augment the available data in our approach, each SMILES representation is converted into graphs, and these graphs are randomized fifty times and used to train the models. This randomization is achieved by swapping the indexes of two atoms in the matrix representation of the graphs. Although we understand that this may create redundancy for small molecules (fewer possible SMILES representations and consequently fewer possible graphs), this will virtually increase the dataset size. This data augmentation ensures that all the molecules are equally represented while also ensuring that any representation of the same molecules would be considered. Evaluation of the impact of this graph randomization and different SMILES for the same molecule will be explored below.

## Supplementary information

Supplementary Material

## Data Availability

All datasets used for training and testing are available at https://github.com/MoitessierLab/pKaLearn. This data release is also linked and citable on Zenodo: 10.5281/zenodo.18691436.

## Code availability

All source code (including training, architecture, and testing scripts) is available at https://github.com/MoitessierLab/pKaLearn. This release is also linked and citable on Zenodo: 10.5281/zenodo.18691436.

## References

[1] Paul, D. et al. Artificial intelligence in drug discovery and development. *Drug Discov. Today*. **26**, 80–93 (2021). 33099022 10.1016/j.drudis.2020.10.010 PMC7577280

[2] Hasselgren, C. & Oprea, T. I. Artificial intelligence for drug discovery: Are we there yet?. *Ann. Rev. Pharmacol. Toxicol.* **64**, 527–550 (2024). 37738505 10.1146/annurev-pharmtox-040323-040828

[3] Yang, X., Wang, Y., Byrne, R., Schneider, G. & Yang, S. Concepts of artificial intelligence for computer-assisted drug discovery. *Chem. Rev.* **119**, 10520–10594 (2019). 31294972 10.1021/acs.chemrev.8b00728

[4] Di Lascio, E., Gerebtzoff, G. & Rodríguez-Pérez, R. Systematic evaluation of local and global machine learning models for the prediction of ADME properties. *Mol. Pharm.* **20**, 1758–1767 (2023). 36745394 10.1021/acs.molpharmaceut.2c00962

[5] Bhhatarai, B., Walters, W. P., Hop, C. E. C. A., Lanza, G. & Ekins, S. Opportunities and challenges using artificial intelligence in ADME/Tox. *Nat. Mater.* **18**, 418–422 (2019). 31000801 10.1038/s41563-019-0332-5 PMC6594826

[6] DeCost, B. L. et al. Scientific AI in materials science: a path to a sustainable and scalable paradigm. *Mach. Learn. Sci. Technol.* **1**, 033001 (2020). 10.1088/2632-2153/ab9a20 PMC7919383 33655211

[7] Pyzer-Knapp, E. O. et al. Accelerating materials discovery using artificial intelligence, high performance computing and robotics. *npj Comput. Mater.* **8**, 84 (2022).

[8] Liu, Y. et al. Generative artificial intelligence and its applications in materials science: current situation and future perspectives. *J. Mater.* **9**, 798–816 (2023).

[9] Bohacek, R. S., McMartin, C. & Guida, W. C. The art and practice of structure-based drug design: a molecular modeling perspective. *Med. Res. Rev.* **16**, 3–50 (1996). 8788213 10.1002/(SICI)1098-1128(199601)16:1<3::AID-MED1>3.0.CO;2-6

[10] Volkov, M. et al. On the frustration to predict binding affinities from protein–ligand structures with deep neural networks. *J. Med. Chem.* **65**, 7946–7958 (2022). 35608179 10.1021/acs.jmedchem.2c00487

[11] Burai Patrascu, M. et al. From desktop to benchtop with automated computational workflows for computer-aided design in asymmetric catalysis. *Nat. Catal.* **3**, 574–584 (2020).

[12] Liu, Z. et al. Elucidating hyperconjugation from electronegativity to predict drug conformational energy in a high throughput manner. *J. Chem. Inf. Model.* **56**, 788–801 (2016). 27028941 10.1021/acs.jcim.6b00012

[13] Liao, C. & Nicklaus, M. C. Comparison of nine programs predicting pKa values of pharmaceutical substances. *J. Chem. Inf. Model.* **49**, 2801–2812 (2009). 19961204 10.1021/ci900289x PMC7289148

[14] Szatylowicz, H. et al. Toward the physical interpretation of inductive and resonance substituent effects and reexamination based on quantum chemical modeling. *ACS Omega* **2**, 7163–7171 (2017). 31457295 10.1021/acsomega.7b01043 PMC6645133

[15] Ugur, I., Marion, A., Parant, S., Jensen, J. H. & Monard, G. Rationalization of the pKa values of alcohols and thiols using atomic charge descriptors and its application to the prediction of amino acid pKa’s. *J. Chem. Inf. Modeling* **54**, 2200–2213 (2014). 10.1021/ci500079w 25089727

[16] Yu, H. S., Watson, M. A. & Bochevarov, A. D. Weighted averaging scheme and local atomic descriptor for pKa prediction based on density functional theory. *J. Chem. Inf. Model.* **58**, 271–286 (2018). 29356524 10.1021/acs.jcim.7b00537

[17] Haslak, Z. P., Zareb, S., Dogan, I., Aviyente, V. & Monard, G. Using atomic charges to describe the pKa of carboxylic acids. *J. Chem. Inf. Model.* **61**, 2733–2743 (2021). 34137248 10.1021/acs.jcim.1c00059

[18] Sandoval-Lira, J., Mondragón-Solórzano, G., Lugo-Fuentes, L. I. & Barroso-Flores, J. Accurate estimation of pKb values for amino groups from surface electrostatic potential (VS,min) calculations: the isoelectric points of amino acids as a case study. *J. Chem. Inf. Model.* **60**, 1445–1452 (2020). 32108480 10.1021/acs.jcim.9b01173

[19] Bochevarov, A. D., Watson, M. A., Greenwood, J. R. & Philipp, D. M. Multiconformation, density functional theory-based pKa prediction in application to large, flexible organic molecules with diverse functional groups. *J. Chem. Theory Comput.* **12**, 6001–6019 (2016). 27951674 10.1021/acs.jctc.6b00805

[20] Thapa, B. & Schlegel, H. B. Density functional theory calculation of pKa’s of thiols in aqueous solution using explicit water molecules and the polarizable continuum model. * J. Phys. Chem. A* **120**, 5726–5735 (2016). 27327957 10.1021/acs.jpca.6b05040

[21] Morency, M., Néron, S., Iftimie, R. & Wuest, J. D. Predicting pKa values of quinols and related aromatic compounds with multiple OH groups. * J. Org. Chem.* **86**, 14444–14460 (2021). 34613729 10.1021/acs.joc.1c01279

[22] Sakti, A. W., Nishimura, Y. & Nakai, H. Rigorous pKa estimation of amine species using density-functional tight-binding-based metadynamics simulations. *J. Chem. Theory Comput.* **14**, 351–356 (2018). 29206463 10.1021/acs.jctc.7b00855

[23] Halstead, S. J., An, P. & Zhang, S. Simulations of dissociation constants in low pressure supercritical water. *Mol. Phys.* **112**, 2235–2240 (2014).

[24] Tummanapelli, A. K. & Vasudevan, S. Estimating successive pKa values of polyprotic acids from ab initio molecular dynamics using metadynamics: the dissociation of phthalic acid and its isomers. *Phys. Chem. Chem. Phys.* **17**, 6383–6388 (2015). 25652329 10.1039/c4cp06000h

[25] Cheng, J., Sulpizi, M. & Sprik, M. Redox potentials and pKa for benzoquinone from density functional theory based molecular dynamics. * J. Chem. Phys.* **131**, 154504 (2009). 20568869 10.1063/1.3250438

[26] Pracht, P., Bohle, F. & Grimme, S. Automated exploration of the low-energy chemical space with fast quantum chemical methods. *Phys. Chem. Chem. Phys.* **22**, 7169–7192 (2020). 32073075 10.1039/c9cp06869d

[27] Bannwarth, C., Ehlert, S. & Grimme, S. GFN2-xTB—an accurate and broadly parametrized self-consistent tight-binding quantum chemical method with multipole electrostatics and density-dependent dispersion contributions. *J. Chem. Theory Comput.* **15**, 1652–1671 (2019). 30741547 10.1021/acs.jctc.8b01176

[28] Abarbanel, O. D. & Hutchison, G. R. QupKake: integrating machine learning and quantum chemistry for micro-pK(a) predictions. *J. Chem. Theory Comput.*10.1021/acs.jctc.4c00328 (2024). 10.1021/acs.jctc.4c00328 PMC11325546 38832803

[29] Meng, Y., Sabri Dashti, D. & Roitberg, A. E. Computing alchemical free energy differences with Hamiltonian Replica Exchange Molecular Dynamics (H-REMD) simulations. *J. Chem. Theory Comput.* **7**, 2721–2727 (2011). 22125475 10.1021/ct200153u PMC3223983

[30] Swails, J. M. & Roitberg, A. E. Enhancing conformation and protonation state sampling of hen egg white lysozyme using pH Replica Exchange Molecular Dynamics. *J. Chem. Theory Comput.* **8**, 4393–4404 (2012). 26605601 10.1021/ct300512h

[31] Olsson, M. H. M., Søndergaard, C. R., Rostkowski, M. & Jensen, J. H. PROPKA3: consistent treatment of internal and surface residues in empirical pKa predictions. *J. Chem. Theory Comput.* **7**, 525–537 (2011). 26596171 10.1021/ct100578z

[32] Reis, P. B. P. S., Vila-Viçosa, D., Rocchia, W. & Machuqueiro, M. PypKa: a flexible Python module for Poisson–Boltzmann-based pKa calculations. *J. Chem. Inf. Model.* **60**, 4442–4448 (2020). 32857502 10.1021/acs.jcim.0c00718

[33] Wang, L., Zhang, M. & Alexov, E. DelPhiPKa web server: predicting pKa of proteins, RNAs and DNAs. *Bioinformatics* **32**, 614–615 (2015). 26515825 10.1093/bioinformatics/btv607 PMC5963359

[34] Anandakrishnan, R., Aguilar, B. & Onufriev, A. V. H++ 3.0: automating pK prediction and the preparation of biomolecular structures for atomistic molecular modeling and simulations. *Nucleic Acids Res.* **40**, W537–W541 (2012). 22570416 10.1093/nar/gks375 PMC3394296

[35] Gunner, M. R. & Baker, N. A. Continuum electrostatics approaches to calculating pKas and Ems in proteins. *Methods Enzymol.* **578**, 1–20 (2016). 27497160 10.1016/bs.mie.2016.05.052 PMC5380367

[36] Song, Y., Mao, J. & Gunner, M. R. MCCE2: improving protein pKa calculations with extensive side chain rotamer sampling. *J. Comput. Chem.* **30**, 2231–2247 (2009). 19274707 10.1002/jcc.21222 PMC2735604

[37] Mansouri, K. et al. Open-source QSAR models for pKa prediction using multiple machine learning approaches. *J. Cheminform.* **11**, 60 (2019). 33430972 10.1186/s13321-019-0384-1 PMC6749653

[38] Baltruschat, M. & Czodrowski, P. Machine learning meets pKa. *F1000Res.* **9**, Chem Inf Sci-113 10.12688/f1000research.22090.2 (2020). 10.12688/f1000research.22090.1 PMC7096188 32226607

[39] Lawler, R. et al. DFT-machine learning approach for accurate prediction of pKa. * J. Phys. Chem. A* **125**, 8712–8722 (2021). 34554744 10.1021/acs.jpca.1c05031

[40] Li, M., Zhang, H., Chen, B., Wu, Y. & Guan, L. Prediction of pKa values for neutral and basic drugs based on hybrid artificial intelligence methods. *Sci. Rep.* **8**, 3991 (2018). 29507318 10.1038/s41598-018-22332-7 PMC5838250

[41] Fraczkiewicz, R. et al. Best of both worlds: combining pharma data and state of the art modeling technology to improve in silico pKa prediction. *J. Chem. Inf. Model.* **55**, 389–397 (2015). 25514239 10.1021/ci500585w

[42] Yang, Q. et al. Holistic prediction of the pKa in diverse solvents based on a machine-learning approach. *Angew. Chem.* **132**, 19444–19453 (2020). 10.1002/anie.202008528 32673431

[43] Hunt, P. et al. Predicting pKa using a combination of semi-empirical quantum mechanics and radial basis function methods. *J. Chem. Inf. Model.* **60**, 2989–2997 (2020). 32357002 10.1021/acs.jcim.0c00105

[44] Xiong, J. et al. Multi-instance learning of graph neural networks for aqueous pKa prediction. *Bioinformatics* **38**, 792–798 (2021). 10.1093/bioinformatics/btab714 PMC8756178 34643666

[45] Kalliokoski, T. & Sinervo, K. Predicting pK(a) for small molecules on public and in-house datasets using fast prediction methods combined with data fusion. *Mol. Inf.* **38**, e1800163 (2019). 10.1002/minf.201800163 31070288

[46] Durant, J. L., Leland, B. A., Henry, D. R. & Nourse, J. G. Reoptimization of MDL keys for use in drug discovery. *J. Chem. Inf. Comput. Sci.* **42**, 1273–1280 (2002). 12444722 10.1021/ci010132r

[47] Rogers, D. & Hahn, M. Extended-connectivity fingerprints. *J. Chem. Inf. Model.* **50**, 742–754 (2010). 20426451 10.1021/ci100050t

[48] Morgan, H. L. The generation of a unique machine description for chemical structures—a technique developed at chemical abstracts service. *J. Chem. Doc.* **5**, 107–113 (1965).

[49] Hall, L. H. & Kier, L. B. Electrotopological state indices for atom types: a novel combination of electronic, topological, and valence state information. *J. Chem. Inf. Comput. Sci.* **35**, 1039–1045 (1995).

[50] Yang, J.-D., Xue, X.-S., Ji, P., Li, X. & Cheng, J.-P. Internet Bondenergy Databank (pKa and BDE) iBonD. http://ibond.chem.tsinghua.edu.cn.

[51] Işık, M. et al. Overview of the SAMPL6 pKa challenge: evaluating small molecule microscopic and macroscopic pKa predictions. *J. Comput. Aided Mol. Des.* **35**, 131–166 (2021). 33394238 10.1007/s10822-020-00362-6 PMC7904668

[52] Pan, X., Wang, H., Li, C., Zhang, J. Z. H. & Ji, C. MolGpka: a web server for small molecule pKa prediction using a graph-convolutional neural network. *J. Chem. Inf. Model.* **61**, 3159–3165 (2021). 34251213 10.1021/acs.jcim.1c00075

[53] Lu, Y. et al. Prediction of pKa using machine learning methods with rooted topological torsion fingerprints: application to aliphatic amines. *J. Chem. Inf. Model.* **59**, 4706–4719 (2019). 31647238 10.1021/acs.jcim.9b00498

[54] Johnston, R. C. et al. Epik: pKa and protonation state prediction through machine learning. *J. Chem. Theory Comput.* **19**, 2380–2388 (2023). 37023332 10.1021/acs.jctc.3c00044

[55] Heid, E. et al. Chemprop: a machine learning package for chemical property prediction. *J. Chem. Inf. Model.* **64**, 9–17 (2024). 38147829 10.1021/acs.jcim.3c01250 PMC10777403

[56] Yang, K. et al. Analyzing learned molecular representations for property prediction. *J. Chem. Inf. Model.* **59**, 3370–3388 (2019). 31361484 10.1021/acs.jcim.9b00237 PMC6727618

[57] Bahr, M. N. et al. Automated high throughput pKa and distribution coefficient measurements of pharmaceutical compounds for the SAMPL8 blind prediction challenge. *J. Comput. Aided Mol. Des.* **35**, 1141–1155 (2021). 34714468 10.1007/s10822-021-00427-0 PMC9313606

[58] Tielker, N. et al. The euroSAMPL1 pKa blind prediction and reproducible research data management challenge. *Phys. Chem. Chem. Phys.* **27**, 18855–18869 (2025). 40891233 10.1039/d5cp01448d

[59] Xiong, Z. et al. Pushing the boundaries of molecular representation for drug discovery with the graph attention mechanism. *J. Med. Chem.* **63**, 8749–8760 (2020). 31408336 10.1021/acs.jmedchem.9b00959

[60] Shi, Y. H. et al. Masked Label Prediction: Unified Message Passing Model for Semi-Supervised Classification. *Proceedings of the Thirtieth International Joint Conference on Artificial Intelligence,* 1548–1554 (2021). 10.24963/ijcai.2021/214.

[61] Luo, W. et al. Bridging machine learning and thermodynamics for accurate pKa prediction. *JACS Au* **4**, 3451–3465 (2024). 39328749 10.1021/jacsau.4c00271 PMC11423309

[62] Zheng, J. & Lafontant-Joseph, O. *IUPAC pKa Data Digitization Report* (MIT, 2025).

[63] Chemical_Book. https://www.chemicalbook.com/ProductMSDSDetailCB8259608_EN.htm (2025).

[64] CookeChem. https://www.cookechem.com/Detail/A4989212.htm (2025).

[65] Watson, M. A., Yu, H. S. & Bochevarov, A. D. Generation of tautomers using micro-pKa’s. *J. Chem. Inf. Model.* **59**, 2672–2689 (2019). 31070917 10.1021/acs.jcim.8b00955

[66] Wahl, O. & Sander, T. Tautobase: an open tautomer database. *J. Chem. Inf. Model.* **60**, 1085–1089 (2020). 31967818 10.1021/acs.jcim.0c00035

[67] Weiser, B., Genzling, J., Burai-Patrascu, M., Rostaing, O. & Moitessier, N. Machine learning-augmented docking. 1. CYP inhibition prediction. *Dig. Discov.* **2**, 1841–1849 (2023).

[68] Roszak, R., Beker, W., Molga, K. & Grzybowski, B. A. Rapid and accurate prediction of pKa values of C–H acids using graph convolutional neural networks. *J. Am. Chem. Soc.* **141**, 17142–17149 (2019). 31633925 10.1021/jacs.9b05895

[69] Gilmer, J., Schoenholz, S. S., Riley, P. F., Vinyals, O. & Dahl, G. E. Neural message passing for quantum chemistry. In *Proc. International Conference on Machine Learning* 1263–1272 (PMLR, 2017).

[70] El-Samman, A. M., De Castro, S., Morton, B. & De Baerdemacker, S. Transfer learning graph representations of molecules for pKa, 13C-NMR, and solubility. *Can. J. Chem.* **102**, 275–288 (2024).

[71] Brody, S. A., U. & Yahav, E. How attentive are graph attention networks?. arXiv preprint *arXiv:2105.14491*. https://openreview.net/pdf?id=F72ximsx7C1 (2021).

[72] RDKit: open-source cheminformatics. https://www.rdkit.org (2025).

[73] Fey, M. & Lenssen, J. E. Fast graph representation learning with PyTorch Geometric. Preprint at *arXiv*10.48550/arXiv.1903.02428 (2019).

[74] Paszke, A. et al. PyTorch: an imperative style, high-performance deep learning library. In *Proc. Advances in Neural Information Processing Systems* Vol. 32 (NeurIPS Proceedings, 2019). https://proceedings.neurips.cc/paper/2019/hash/bdbca288fee7f92f2bfa9f7012727740-Abstract.html.

[75] Pedregosa, F. et al. Scikit-learn: machine learning in Python. * J. Mach. Learn. Res.* **12**, 2825–2830 (2011).

[76] Arús-Pous, J. et al. Randomized SMILES strings improve the quality of molecular generative models. *J. Cheminform.* **11**, 71 (2019). 33430971 10.1186/s13321-019-0393-0 PMC6873550

[77] Kikuchi, Y. et al. Impact of SMILES notational inconsistencies on chemical language models trained via molecular translation. Preprint at *arXiv*10.48550/arXiv.2505.07139 (2026).

[78] Mayr, F., Wieder, M., Wieder, O. & Langer, T. Improving small molecule pKa prediction using transfer learning with graph neural networks. *Front. Chem*. **10**, 10.3389/fchem.2022.866585 (2022). 10.3389/fchem.2022.866585 PMC9204323 35721000

[79] Diedenhofen, M., Eckert, F. & Terzi, S. COSMO-RS blind prediction of distribution coefficients and aqueous pKa values from the SAMPL8 challenge. *J. Comput. Aided Mol. Des.* **37**, 395–405 (2023). 37365370 10.1007/s10822-023-00514-4

