# Supplementary material for: Development of a pKa predictor (pKaLearn) by leveraging teaching experience to improve machine learning
Source: Commun Chem. 2026 Mar 26;9:181. doi: 10.1038/s42004-026-01983-y (PMC13183876; doi:10.1038/s42004-026-01983-y)
Supplement: Supplementary file 1 — Supplementary Material [file 42004_2026_1983_MOESM1_ESM.pdf]

Development of a pKa predictor (pKaLearn) by leveraging teaching experience to improve machine learning.

Jérôme Genzling, Ziling Luo, Benjamin Weiser, Nicolas Moitessier*

Department of Chemistry, McGill University, 801 Sherbrooke St W, Montreal, QC, Canada H3A 0B8
* nicolas.moitessier@mcgill.ca

Contents

S-I. Analysis of experimental data. 2
S-II. Supplementary Note 1. 2
S-III. Supplementary Note 2 4
S-III.1. Graph Attention Network (GAT) - Schematic representation 4
S-III.2. Theory 5
S-III.3. Encoding 6
S-IV. Conjugation 7
S-V. Results 8
S-VI. Code and data availability 21
S-VII. Supplementary References 21

S-I. Analysis of experimental data.

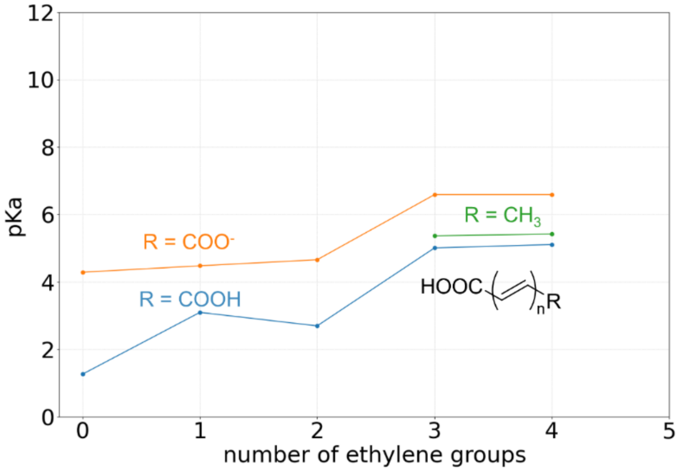

Supplementary Figure 1. pKa and resonance effect.

S-II. Supplementary Note 1.

Dataset. Assembling a high-quality dataset is a great challenge. Reported pKa measurements used to train model for pKa prediction are no exception. This pKa data may be measured using for example potentiometric methods or NMR spectroscopy (or even quantum mechanics[83]). As discussed by Chodera and co-workers,[84] this leads to pKa being either macroscopic or microscopic pKa. These differences could then lead to errors in the data reported in the various pKa datasets collected. For instance, the molecules shown in Supplementary Figure 1 were reported with significant pKa differences (higher than 0.25 units of pKa). We believe that these differences may come from various error sources, either experimental or in the reported datasets (wrong value, wrong SMILES -wrong protonation state - encoding of the molecule tested). The "Error" columns in the dataset quantify the variability in pKa values for the same molecule across different sets. For single-source values, the pKa is taken directly, with an error of 0. For two sources, the pKa value is the mean of the values, and the error is half the absolute difference between the two pKa values. For three sources, the pKa is the mean, and the error is the maximum of the differences between the mean and the extreme value. Large error values indicate molecules with significant discrepancies, warranting individual examination. These corrections enhance the reliability of the error calculations and the overall dataset integrity.

Some errors are also difficult to detect. For example, according to Suschitzky and Sheard "a pKa value of 8.9 has been claimed for chromone-3-carboxylic acids but we have determined the parent compound (R=H) to have a more likely of 4.0 using conductometric method".[86] In fact, chromone-3-carboxylic acid was a clear outlier in our predictions when a pKa of 8.9 was used. In addition, in some available databases, the ionization center is labeled but, as the source of each value is provided, it is difficult to assess whether it was assigned experimentally (e.g., using NMR spectroscopy) or as an informed guess. As microstates may co-exist, a single protonation state is not the correct representation of a molecule. This problem will be addressed using microstates with assigned ionization centers (single protonation state) in the training set teaching the model the pKa for this site (regardless of other microstates). However, the testing will be carried out on multiple microstates for which the pKa of the most basic site will be predicted. This strategy simulates the titration of each site one after the other.

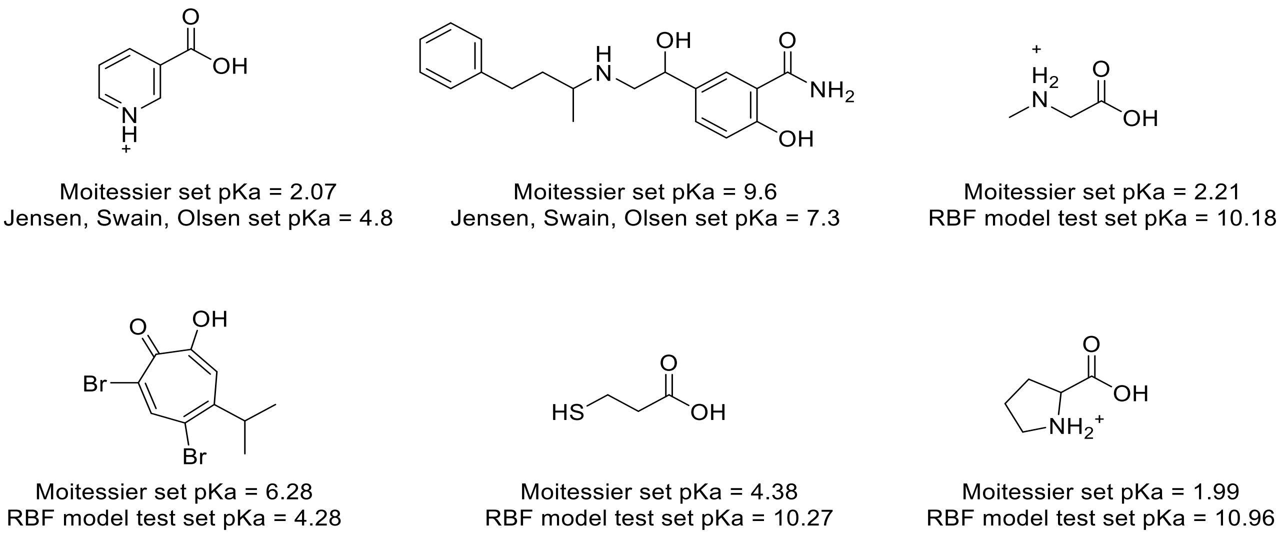

Supplementary Figure 2. Examples of large pKa differences collected during the cleaning of the various sets. Here are examples of molecules collected from our in-house set build over the years (Experimental papers, DataWarrior and ChEMBL) compared to the sets provided by Hunt et *al.*[84]These values were corrected using cross validation from other sources and our chemistry knowledge**.**

Our dataset collection relied on three datasets. The first one is an in-house dataset of experimental pKa values collected over the years from various experimental sections of the literature as well as public databases such as ChEMBL[87] and DataWarrior (https://openmolecules.org/datawarrior/datafiles.html) datasets. We also included the sets provided by Baltruschat et *al.*[88] and Hunt et *al.*[85] as well as the molecules from the SAMPL6 and 7 blind challenges.[89,90] To ensure high quality of the final dataset, each of the original data points was first standardized by the RDKit workflow followed by rigorously checking correct ionization states and pKa values. Although tedious, this step was believed to be essential for optimal accuracy of our models. Four principles were followed when curating each pKa data: a) pKa data must be measured by experimental techniques instead of determined by computational methods to maintain high accuracy of the dataset; b) Considering the solvent effect is prominent when measuring pKa, each data must be labelled with water as solvent and in the range of (-1.0, 15.0); c) For compounds with several recorded data either from different references or measured by different techniques, the most reliable pKa (e.g., cross validation by different techniques) data with correct ionization states were selected; d) pKa data without appropriate labelling (solvents, techniques, or references) were removed. To maintain a single and uniform dataset, pKa data labelled as bases from DataWarrior were converted into their conjugate acid. Our pipeline also includes steps to remove duplicate molecules even if provided with different SMILES representation. The final set includes 12,757 molecules.

Training and testing sets. Selecting data points for training and testing sets must be done with care to avoid overlap between the two. In particular, when splitting randomly, analogues may appear in both sets. In the latter case, if the model is overtrained, the accuracy on the testing set may be overestimated and the overtraining undetected. Following our recently reported strategy to reduce this overlap,[91] we split the dataset into training and testing sets ensuring a max Tanimoto coefficient (similarity measurement) between every pair of molecules (one from the training set and one from the testing set) of 0.65 using ECFP4 fingerprint.

S-III. Supplementary Note 2

S-III.1. Graph Attention Network (GAT) - Schematic representation

Supplementary Figure 3 is illustrating the principle of graph and how the features were attributed to atoms and bonds. In this figure, we also illustrate how masks were applied. When the mask is applied (atoms within 2 bonds in Supplementary Figure 3), all the atoms and bonds outside the mask are ignored and only the fragment around the ionization center is considered.

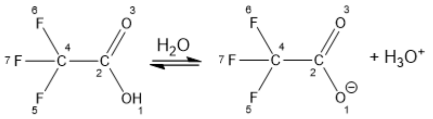

Atom features

	B	C	N	O	F	Si	P	S*	Cl	Br	I	χ	η	r	hybridization						A		rings			Ring size			Hydrogens						FC						IC
1	0	0	0	1	0	0	0	0	0	0	0	D	D	D	0	0	1	0	0	1	0	0	0	0	0	0	0	0	0	1	0	1	0	0	0	1	0	1	0	0	1
2	0	1	0	0	0	0	0	0	0	0	0	D	D	D	0	1	0	0	1	0	0	0	0	0	0	0	0	0	1	0	0	1	0	0	0	1	0	0	1	0	0
3	0	0	0	1	0	0	0	0	0	0	0	D	D	D	0	1	0	0	1	0	0	0	0	0	0	0	0	0	1	0	0	1	0	0	0	1	0	0	1	0	0
4	0	1	0	0	0	0	0	0	0	0	0	D	D	D	0	0	1	0	0	1	0	0	0	0	0	0	0	0	1	0	0	1	0	0	0	1	0	0	1	0	0
5	0	0	0	0	1	0	0	0	0	0	0	D	D	D	0	0	1	0	0	1	0	0	0	0	0	0	0	0	1	0	0	1	0	0	0	1	0	0	1	0	0
6	0	0	0	0	1	0	0	0	0	0	0	D	D	D	0	0	1	0	0	1	0	0	0	0	0	0	0	0	1	0	0	1	0	0	0	1	0	0	1	0	0
7	0	0	0	0	1	0	0	0	0	0	0	D	D	D	0	0	1	0	0	1	0	0	0	0	0	0	0	0	1	0	0	1	0	0	0	1	0	0	1	0	0

S*: S or Se, χ: normalized electronegativity, η: normalized hardness, r: normalized atomic radius, hybridization: sp, sp2, sp3 for acid and base, A: aromaticity (acid and base), Rings: no ring, 1 ring, 2+ rings, Ring size: 3, 4, 5, 6+, Hydrogens: no H, 1 H, 2H's, 3+ H's, FC: formal charge -1, 0, 1 for acid and base, IC: ionization center or not.
D stands for decimal (not only 0 or 1)

Adjacency matrix with edge features.

	1	2	3	4	5	6	7
1	0 0 0 0 0 0 0 0 0 0 0.0 0.0	1 0 0 0 0 1 0 0 0 1 D D	0 0 0 0 0 0 0 0 0 0 0.0 0.0	0 0 0 0 0 0 0 0 0 0 0.0 0.0	0 0 0 0 0 0 0 0 0 0 0.0 0.0	0 0 0 0 0 0 0 0 0 0 0.0 0.0	0 0 0 0 0 0 0 0 0 0 0.0 0.0
2	1 0 0 0 0 1 0 0 0 1 D D	0 0 0 0 0 0 0 0 0 0 0.0 0.0	0 0 1 0 0 0 1 0 0 1 D D	1 0 0 0 1 0 0 0 0 0 D D	0 0 0 0 0 0 0 0 0 0 0.0 0.0	0 0 0 0 0 0 0 0 0 0 0.0 0.0	0 0 0 0 0 0 0 0 0 0 0.0 0.0
3	0 0 0 0 0 0 0 0 0 0 0.0 0.0	0 0 1 0 0 0 1 0 0 1 D D	0 0 0 0 0 0 0 0 0 0 0.0 0.0	0 0 0 0 0 0 0 0 0 0 0.0 0.0	0 0 0 0 0 0 0 0 0 0 0.0 0.0	0 0 0 0 0 0 0 0 0 0 0.0 0.0	0 0 0 0 0 0 0 0 0 0 0.0 0.0
4	0 0 0 0 0 0 0 0 0 0 0.0 0.0	1 0 0 0 1 0 0 0 0 0 D D	0 0 0 0 0 0 0 0 0 0 0.0 0.0	0 0 0 0 0 0 0 0 0 0 0.0 0.0	1 0 0 0 1 0 0 0 0 0 D D	1 0 0 0 1 0 0 0 0 0 D D	1 0 0 0 1 0 0 0 0 0 D D
5	0 0 0 0 0 0 0 0 0 0 0.0 0.0	0 0 0 0 0 0 0 0 0 0 0.0 0.0	0 0 0 0 0 0 0 0 0 0 0.0 0.0	1 0 0 0 1 0 0 0 0 0 D D	0 0 0 0 0 0 0 0 0 0 0.0 0.0	0 0 0 0 0 0 0 0 0 0 0.0 0.0	0 0 0 0 0 0 0 0 0 0 0.0 0.0
6	0 0 0 0 0 0 0 0 0 0 0.0 0.0	0 0 0 0 0 0 0 0 0 0 0.0 0.0	0 0 0 0 0 0 0 0 0 0 0.0 0.0	1 0 0 0 1 0 0 0 0 0 D D	0 0 0 0 0 0 0 0 0 0 0.0 0.0	0 0 0 0 0 0 0 0 0 0 0.0 0.0	0 0 0 0 0 0 0 0 0 0 0.0 0.0
7	0 0 0 0 0 0 0 0 0 0 0.0 0.0	0 0 0 0 0 0 0 0 0 0 0.0 0.0	0 0 0 0 0 0 0 0 0 0 0.0 0.0	1 0 0 0 1 0 0 0 0 0 D D	0 0 0 0 0 0 0 0 0 0 0.0 0.0	0 0 0 0 0 0 0 0 0 0 0.0 0.0	0 0 0 0 0 0 0 0 0 0 0.0 0.0

Each cell represents: bond order (single, aromatic, double, triple) for acid and base, conjugation for acid and base, polarization for acid and base (represented as the difference of normalized electronegativities between the two connected atoms, with the first one being the one closer to the ionization center.
D stands for decimal (not only 0 or 1)

Supplementary Figure 3. Atom and bond features. With a mask of two, all the atoms further than 2 bonds away are ignored. D stands for decimals normalized to the interval [0, 1].

As illustrated in Supplementary Figure 4, the stacking on GAT layers allows the ionization center (O_1) to have some carbonyl carbon flavor (C_2) after the first iteration. C_4 will also have some fluorine (F_5, F_6 and F_7) characteristics. As a result, a mask of 2 (from O_1 as far as C_4) will include the electronegativity effects of fluorine atoms even if the fluorine atoms are not considered in the mask. A GAT layer and a mask of size 2 consider the effect of atoms as far as three bonds away. Similarly, after a second GAT layer, the fluorine features are captured by C_2, one bond away from the ionization center O_1. Two GAT layers and a mask of size 2 considers atoms as far as four bonds away.

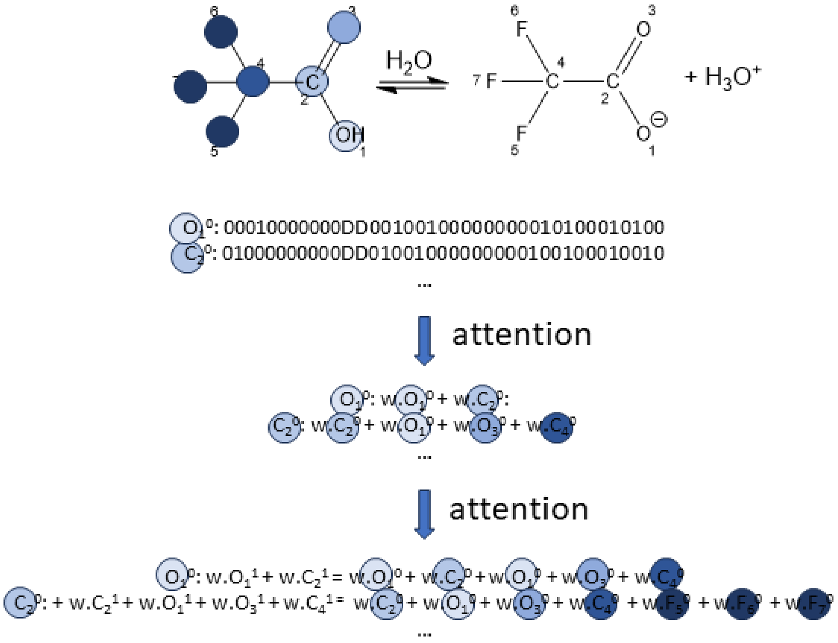

Supplementary Figure 4. Atom and bond features. With a mask of two, all the atoms further than 2 bonds away are ignored. With w being weights (optimized through training).

S-III.2. Theory

A single GNN layer, which is the foundation of the GNN model, updates each node by aggregating information from its directly connected neighbours. As a result, the final nodes collect messages from the local environment of the graph by stacking multiple GNN layers. The main difference between different types of GNN models is the aggregation strategy when passing messages from neighbours. Each neighbour of node v_i contributes isotropically in the graph convolutional network (GCN)[92] layers, as shown in the equation (1),

$$h_i^{l+1} = \sigma\left(\sum_j \frac{1}{c_{ij}} h_j^l W^l\right) \quad (1)$$

Where h_i represents the state vector of the central node i, j denotes the direct neighbors of the central node, c_{ij} is the normalization constant which defines the isotropic average operator, σ is the activation function and W^l denotes the learnable weight matrix of current layer l. The GCN is conceptually simple and easy to implement but the biggest shortcoming is the message passing approach treats each direct neighbour equally instead of learning their different contributions (e.g., a carbonyl bound to a nitrogen – making up an amide- has more impact than a hydrogen). Graph attention network[93] applies the graph attention mechanism[94] which enables the anisotropic learning during the message passing step. The graph attention mechanism is defined by the following equations,

$$h_i^{l+1} = \sigma\left(\sum_j \alpha_{ij}^l z_j^l\right) \quad (2)$$

where,

$$\alpha_{ij}^l = softmax(\beta_{ij}) = \frac{\exp(\beta_{ij})}{\sum_k \exp(\beta_{ik})} \quad (3)$$

$$\beta_{ij} = LeakyReLU\left(a^{l^T}\left[W^l h_i^l \,||W^l h_j^l \,||W_e^l e_{ij}^l\right]\right) \quad (4)$$

The graph attention mechanism contains three main steps. The state vectors of node i, its direct neighbors, and the edge features are firstly concatenated followed by embedding with learnable weight matrix W ($[W^l h_i^l \,||\, W^l h_j^l \,||\, W_e^l e_{ij}^l]$). The LeakyReLU activation function is implemented after taking the dot product with the learnable attention weigh matrix a, since it performs better by allowing a small portion of extension on the negative side compared with the ReLU function. During the normalization step, the Softmax activation function (Eq.4) is used to measure the attention score or the importance (α_{ij}) of each neighbor of current node. Finally, the features of neighbor nodes are aggregated and scaled by their attention score to the target node i. More recently, an improved version of GAT model[95] has been introduced to avoid collapsing into a single linear layer by adjusting the order of attention weight matrix and LeakyReLU activation function (Eq.5). The performance of the updated model has been reported to have better performance compared with the original algorithm.[95]

$$\beta_{ij} = a^{l^T} LeakyReLU\left(\left[W^l h_i^l \,||W^l h_j^l \,||W_e^l e_{ij}^l\right]\right) \quad (5)$$

Although there are no limitations on the number of layers, GNN models still may suffer the over-smoothing problem when stacking too many GNN layers. Also, self-attention mechanism is not stable in the GAT model with single head. Multi-head attention mechanism which replicates the three steps of self-attention approach multiple times has been proposed to generate one h_i^{l+1} per attention head.[95] The GAT model combined with the multi-head attention mechanism is more stable and expected to be more robust to the over-smoothing problem compared with the self-attention GAT model.

S-III.3. Encoding

We built a graph attention network with multi-head attention mechanism using PyTorch. According to the analysis of inductive and resonance effect, we built GNN models based on the local environment around the ionization center instead of the entire molecules which also enables the pK_a on large organic systems.

The features were the following:

1. The element and its properties are key factors for pKa (e.g., C-H is less acidic than O-H). 11 bits were added for elements (categorical). Selected atomic properties provide a first level of teaching. Three bits to capture atomic electronegativity and hardness (inductive effects) as well as atomic diameter were added (interval).
2. Formal charge is also associated with acidity ($R\text{-}NH_2$ less acidic than $R\text{-}NH_3^+$). Three bits for the formal charge (-1, 0, and 1) for both acid and conjugate base.
3. Hybridization affects pKa (e.g., terminal alkynes are more acidic than alkenes, which are in turn more acidic than alkanes; iminium more acidic than ammonium). Three bits were included for hybridization states: sp, sp^2, sp^3 and one bit for aromaticity for both acid and conjugate base as these properties may change from acid to conjugate base (e.g., **7a/b**).
4. Rings influence pKa (e.g., **9a/b** vs. **10a/b**): four bits for whether the atom is in a ring with a specific size (3, 4, 5, ≥6-membered ring).
5. The local environment also affects pKa. Three bits for the number of hydrogen atoms connected to the ionization center (0, 1, 2 and 3 or 4) for both acid and conjugate base.
6. To direct the training towards the ionization center, one bit was added defining whether the atom is the ionization center or not.
7. The molecular net charge and ionization formal charge are two factors modulating pKa: two bits added to the embedding (after graph layers), which is processed through 2 fully connected layers.

The edge feature matrix is used to represent the bond properties including:

1. Bond type (single, aromatic, double, and triple bond), 4 bits (categorical)
2. Conjugation (categorical)
3. Bond polarization, computed as a difference in electronegativity of the two atoms making up the bond (interval).

The atom numbers are then reorganized to ensure that the ionization center is atom #1.

To derive our optimal model, hyperparameters for the 4-4 model were optimized using the Tree of Parzen Estimator in hyperopt before running the Proof-of-Concept study.[96]

S-IV. Conjugation

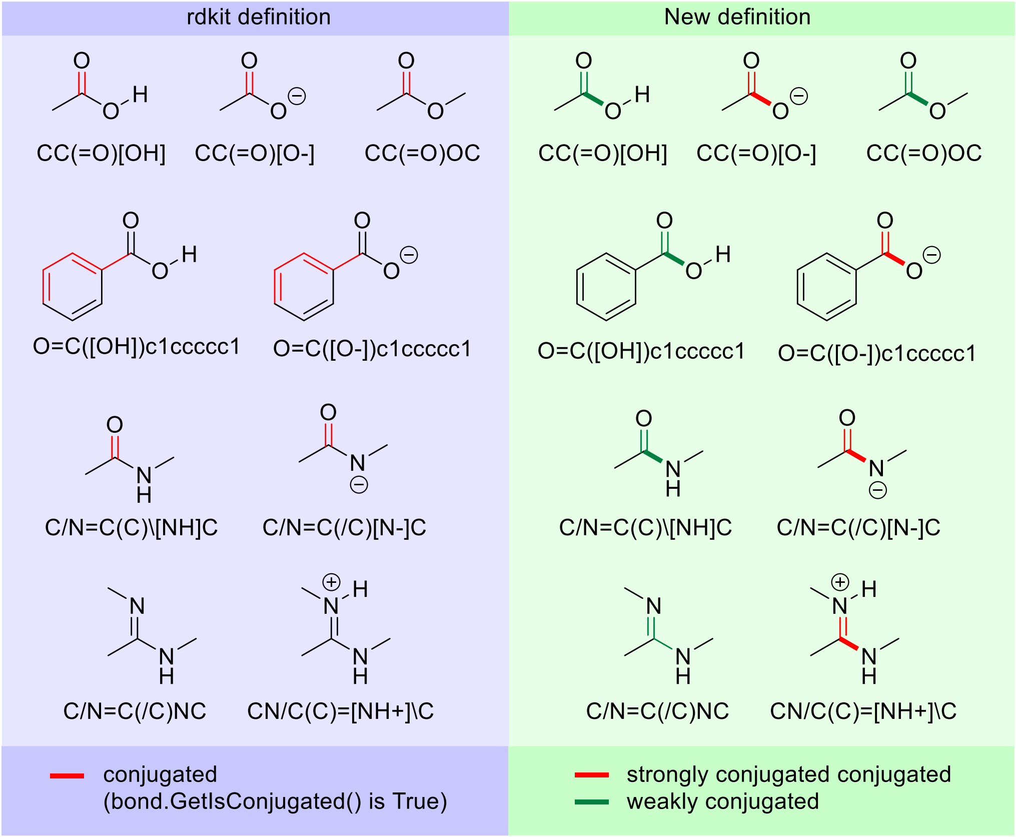

Supplementary Figure 5. Definition of conjugation used for bond features.

S-V. Results

Supplementary Table 1. Numerical results (MAE) for Figure 5

			Training set			Testing set		
N graph layers	Mask size	Label	run#1	run#2	run#3	run#1	run#2	run#3
1	1	1-1	0.899	0.881	0.901	1.007	1.010	1.042
1	2	1-2	0.694	0.687	0.671	0.866	0.854	0.829
2	1	2-1	0.804	0.788	0.811	0.896	0.890	0.901
1	3	1-3	0.513	0.538	0.525	0.772	0.800	0.769
2	2	2-2	0.648	0.616	0.622	0.761	0.777	0.817
3	1	3-1	0.665	0.634	0.703	0.809	0.839	0.821
1	4	1-4	0.489	0.459	0.414	0.762	0.755	0.783
2	3	2-3	0.472	0.498	0.529	0.736	0.758	0.762
3	2	3-2	0.509	0.560	0.572	0.719	0.755	0.751
4	1	4-1	0.588	0.614	0.613	0.776	0.790	0.787
1	5	1-5	0.430	0.437	0.455	0.766	0.774	0.759
2	4	2-4	0.475	0.473	0.431	0.731	0.739	0.720
3	3	3-3	0.473	0.503	0.459	0.700	0.711	0.688
4	2	4-2	0.546	0.540	0.554	0.730	0.718	0.709
5	1	5-1	0.620	0.575	0.533	0.755	0.743	0.742
1	6	1-6	0.415	0.425	0.407	0.765	0.790	0.806
2	5	2-5	0.458	0.427	0.451	0.695	0.710	0.729
3	4	3-4	0.435	0.435	0.442	0.675	0.679	0.694
4	3	4-3	0.515	0.522	0.486	0.708	0.709	0.716
5	2	5-2	0.554	0.476	0.576	0.716	0.699	0.746
6	1	6-1	0.555	0.516	0.573	0.773	0.716	0.750
1	7	1-7	0.420	0.407	0.432	0.768	0.795	0.771
2	6	2-6	0.462	0.451	0.429	0.715	0.721	0.680
3	5	3-5	0.435	0.424	0.413	0.691	0.703	0.678
4	4	4-4	0.464	0.468	0.452	0.679	0.686	0.693
5	3	5-3	0.535	0.472	0.503	0.711	0.709	0.694
6	2	6-2	0.515	0.503	0.525	0.735	0.733	0.737
7	1	7-1	0.508	0.516	0.473	0.762	0.774	0.764

Supplementary Table 2. Numerical results (MAE) for Figure 6

			Training set			Testing set		
N graph layers	Mask size	Label	run#1	run#2	run#3	run#1	run#2	run#3
3	4	1	0.371	0.410	0.389	0.683	0.706	0.705
3	5	1	0.454	0.326	0.395	0.685	0.657	0.676
4	3	1	0.434	0.443	0.417	0.673	0.700	0.671
4	4	1	0.355	0.421	0.434	0.666	0.667	0.664

5	3	1	0.419	0.444	0.429	0.654	0.709	0.685
3	4	2	0.392	0.416	0.382	0.684	0.710	0.687
3	5	2	0.339	0.358	0.394	0.652	0.670	0.705
4	3	2	0.401	0.403	0.372	0.668	0.687	0.690
4	4	2	0.464	0.374	0.377	0.689	0.709	0.695
5	3	2	0.465	0.380	0.335	0.700	0.661	0.665
3	4	3	0.507	0.528	0.497	0.817	0.831	0.807
3	5	3	0.457	0.450	0.468	0.788	0.795	0.790
4	3	3	0.642	0.504	0.542	0.883	0.791	0.799
4	4	3	0.470	0.489	0.445	0.802	0.771	0.784
5	3	3	0.622	0.636	0.632	0.815	0.864	0.849
3	4	4	0.407	0.429	0.422	0.730	0.738	0.726
3	5	4	0.372	0.422	0.383	0.713	0.746	0.708
4	3	4	0.449	0.458	0.413	0.696	0.738	0.756
4	4	4	0.442	0.433	0.435	0.750	0.733	0.679
5	3	4	0.560	0.391	0.458	0.777	0.716	0.757
3	4	5	0.398	0.404	0.432	0.685	0.692	0.701
3	5	5	0.403	0.420	0.380	0.719	0.688	0.674
4	3	5	0.390	0.355	0.361	0.655	0.637	0.653
4	4	5	0.421	0.389	0.330	0.681	0.660	0.645
5	3	5	0.372	0.415	0.409	0.650	0.652	0.660
3	4	6	0.404	0.404	0.412	0.690	0.686	0.701
3	5	6	0.407	0.401	0.399	0.675	0.686	0.672
4	3	6	0.391	0.348	0.394	0.659	0.685	0.672
4	4	6	0.392	0.409	0.385	0.676	0.680	0.668
5	3	6	0.509	0.438	0.487	0.703	0.691	0.687
3	4	7	0.368	0.404	0.425	0.662	0.685	0.693
3	5	7	0.357	0.453	0.387	0.638	0.713	0.669
4	3	7	0.360	0.386	0.383	0.684	0.650	0.662
4	4	7	0.359	0.350	0.355	0.664	0.660	0.673
5	3	7	0.399	0.433	0.438	0.661	0.705	0.701
3	4	8	0.463	0.447	0.408	0.706	0.709	0.683
3	5	8	0.400	0.403	0.390	0.673	0.662	0.708
4	3	8	0.425	0.437	0.506	0.676	0.691	0.702
4	4	8	0.446	0.403	0.477	0.681	0.677	0.697
5	3	8	0.453	0.494	0.371	0.697	0.740	0.644
3	4	9	0.425	0.390	0.386	0.671	0.661	0.697
3	5	9	0.365	0.375	0.403	0.671	0.671	0.664
4	3	9	0.419	0.416	0.438	0.695	0.695	0.689
4	4	9	0.416	0.405	0.379	0.663	0.672	0.660
5	3	9	0.398	0.433	0.504	0.658	0.674	0.670
3	4	10	0.410	0.421	0.380	0.710	0.724	0.687

3	5	10	0.423	0.471	0.448	0.748	0.723	0.723
4	3	10	0.409	0.416	0.478	0.686	0.702	0.729
4	4	10	0.405	0.396	0.436	0.708	0.694	0.726
5	3	10	0.461	0.511	0.493	0.716	0.750	0.748
3	4	11	0.524	0.487	0.483	0.750	0.671	0.704
3	5	11	0.481	0.481	0.518	0.714	0.674	0.741
4	3	11	0.563	0.536	0.595	0.735	0.767	0.735
4	4	11	0.497	0.466	0.500	0.712	0.697	0.722
5	3	11	0.553	0.662	0.496	0.732	0.822	0.702
3	4	12	0.403	0.432	0.445	0.694	0.707	0.723
3	5	12	0.429	0.360	0.409	0.700	0.665	0.690
4	3	12	0.385	0.448	0.445	0.657	0.686	0.679
4	4	12	0.440	0.505	0.408	0.699	0.707	0.671
5	3	12	0.376	0.429	0.428	0.651	0.654	0.660
3	4	13	0.469	0.445	0.360	0.733	0.701	0.665
3	5	13	0.421	0.430	0.428	0.730	0.705	0.695
4	3	13	0.468	0.479	0.458	0.713	0.764	0.724
4	4	13	0.412	0.453	0.432	0.721	0.711	0.700
5	3	13	0.494	0.465	0.551	0.735	0.723	0.807
3	4	14	0.344	0.334	0.328	0.668	0.643	0.645
3	5	14	0.353	0.324	0.355	0.660	0.635	0.611
4	3	14	0.309	0.325	0.327	0.635	0.645	0.657
4	4	14	0.333	0.343	0.377	0.656	0.631	0.678
5	3	14	0.324	0.314	0.356	0.625	0.618	0.643
3	4	15	0.409	0.413	0.406	0.741	0.746	0.731
3	5	15	0.394	0.403	0.379	0.730	0.720	0.737
4	3	15	0.430	0.487	0.447	0.709	0.750	0.697
4	4	15	0.409	0.367	0.388	0.710	0.718	0.711
5	3	15	0.459	0.483	0.436	0.733	0.732	0.750
3	4	16	0.423	0.424	0.418	0.666	0.716	0.689
3	5	16	0.429	0.465	0.361	0.703	0.731	0.654
4	3	16	0.392	0.437	0.436	0.662	0.697	0.656
4	4	16	0.405	0.390	0.409	0.676	0.665	0.701
5	3	16	0.471	0.366	0.424	0.682	0.660	0.645
3	4	17	0.405	0.434	0.404	0.684	0.698	0.704
3	5	17	0.433	0.379	0.398	0.701	0.660	0.697
4	3	17	0.391	0.415	0.447	0.669	0.689	0.673
4	4	17	0.369	0.377	0.346	0.660	0.636	0.655
5	3	17	0.414	0.412	0.449	0.690	0.658	0.661
3	4	18	0.339	0.346	0.329	0.675	0.672	0.730
3	5	18	0.262	0.304	0.248	0.614	0.628	0.613
4	3	18	0.288	0.278	0.28	0.662	0.625	0.689

4	4	18	0.306	0.363	0.314	0.643	0.742	0.673
5	3	18	0.247	0.361	0.317	0.630	0.69	0.663
3	4	19	0.263	0.264	0.332	0.613	0.62	0.613
3	5	19	0.309	0.320	0.305	0.626	0.65	0.619
4	3	19	0.343	0.332	0.362	0.621	0.618	0.665
4	4	19	0.289	0.307	0.251	0.645	0.705	0.635
5	3	19	0.383	0.347	0.316	0.672	0.663	0.635
3	4	20	0.349	0.307	0.403	0.648	0.635	0.731
3	5	20	0.339	0.365	0.319	0.652	0.658	0.629
4	3	20	0.369	0.351	0.449	0.661	0.627	0.727
4	4	20	0.380	0.341	0.350	0.670	0.670	0.644
5	3	20	0.308	0.308	0.320	0.624	0.630	0.623
3	4	21	0.310	0.270	0.296	0.611	0.615	0.617
3	5	21	0.301	0.285	0.308	0.605	0.628	0.640
4	3	21	0.289	0.294	0.359	0.644	0.640	0.634
4	4	21	0.316	0.266	0.285	0.636	0.632	0.637
5	3	21	0.309	0.333	0.319	0.645	0.633	0.600
3	4	22	0.346	0.397	0.365	0.658	0.680	0.683
3	5	22	0.321	0.300	0.424	0.676	0.644	0.689
4	3	22	0.383	0.405	0.353	0.663	0.689	0.636
4	4	22	0.353	0.392	0.366	0.667	0.713	0.630
5	3	22	0.300	0.293	0.391	0.628	0.618	0.662
3	4	23	0.282	0.330	0.283	0.625	0.666	0.639
3	5	23	0.279	0.260	0.243	0.653	0.605	0.618
4	3	23	0.269	0.294	0.275	0.625	0.638	0.654
4	4	23	0.287	0.279	0.253	0.641	0.664	0.640
5	3	23	0.313	0.276	0.287	0.626	0.661	0.622
3	4	24	0.216	0.227	0.245	0.521	0.498	0.542
3	5	24	0.197	0.213	0.199	0.506	0.496	0.498
4	3	24	0.240	0.273	0.254	0.570	0.538	0.572
4	4	24	0.197	0.228	0.212	0.521	0.494	0.505
5	3	24	0.212	0.221	0.203	0.548	0.507	0.523

Supplementary Table 3. Numerical results (MAE) for our pKaLearn models in Table 1 with ionization center (IC) provided (yes) or not (no). 4 graph layers and a mask size of 4 as well as for Chemprop and MolGpKa.

			Training set			Testing set		
GNN	IC	Entry	run#1	run#2	run#3	run#1	run#2	run#3
GATv2Conv	No	1	0.282	0.297	0.250	0.648	0.654	0.655
GATv2Conv	No	1	0.240	0.281	0.273	0.614	0.615	0.620
GATv2Conv	Yes	1	0.165	0.164	0.121	0.620	0.606	0.599
GATv2Conv	Yes	1	0.145	0.172	0.167	0.594	0.587	0.587

GATv2Conv	No	2	0.295	0.286	0.212	0.559	0.585	0.590
GATv2Conv	No	2	0.309	0.334	0.273	0.593	0.578	0.622
GATv2Conv	Yes	2	0.220	0.199	0.121	0.579	0.590	0.591
GATv2Conv	Yes	2	0.241	0.270	0.187	0.578	0.578	0.602
GATv2Conv	No	3	0.218	0.333	0.300	0.719	0.676	0.670
GATv2Conv	No	3	0.333	0.232	0.352	0.733	0.725	0.752
GATv2Conv	Yes	3	0.136	0.277	0.245	0.726	0.759	0.705
GATv2Conv	Yes	3	0.263	0.156	0.284	0.791	0.770	0.800
GATv2Conv	No	4	0.190	0.512	0.499	0.668	0.649	0.691
GATv2Conv	No	4	0.395	0.272	0.290	0.631	0.697	0.699
GATv2Conv	Yes	4	0.103	0.480	0.273	0.702	0.717	0.667
GATv2Conv	Yes	4	0.357	0.210	0.233	0.685	0.713	0.734
GATv2Conv	No	5	0.239	0.284	0.168	0.440	0.394	0.396
GATv2Conv	No	5	0.206	0.228	0.248	0.404	0.385	0.369
GATv2Conv	Yes	5	0.166	0.224	0.087	0.425	0.402	0.380
GATv2Conv	Yes	5	0.124	0.145	0.169	0.380	0.401	0.369
GATv2Conv	No	6	0.164	0.261	0.233	0.331	0.349	0.365
GATv2Conv	No	6	0.198	0.277	0.263	0.312	0.366	0.319
GATv2Conv	Yes	6	0.078	0.183	0.144	0.306	0.330	0.328
GATv2Conv	Yes	6	0.119	0.217	0.187	0.289	0.355	0.298
GATv2Conv	No	7	0.244	0.213	0.224	0.499	0.480	0.470
GATv2Conv	No	7	0.175	0.254	0.188	0.488	0.484	0.479
GATv2Conv	Yes	7	0.304	0.132	0.152	0.499	0.489	0.489
GATv2Conv	Yes	7	0.082	0.168	0.110	0.486	0.493	0.487
AttentiveFP	No	1	0.341	0.748	0.334	0.709	0.328	0.709
AttentiveFP	No	1	0.323	0.688	0.294	0.706	0.353	0.750
AttentiveFP	Yes	1	0.311	0.701	0.371	0.708	0.387	0.713
AttentiveFP	Yes	1	0.236	0.681	0.231	0.680	0.224	0.682
AttentiveFP	No	2	0.234	0.651	0.197	0.670	0.248	0.703
AttentiveFP	No	2	0.217	0.676	0.293	0.688	0.288	0.688
AttentiveFP	Yes	2	0.260	0.663	0.287	0.675	0.370	0.686
AttentiveFP	Yes	2	0.274	0.647	0.364	0.640	0.304	0.655
AttentiveFP	No	3	0.325	0.704	0.298	0.648	0.342	0.638
AttentiveFP	No	3	0.161	0.660	0.195	0.671	0.267	0.679
AttentiveFP	Yes	3	0.189	0.657	0.303	0.641	0.221	0.653
AttentiveFP	Yes	3	0.204	0.645	0.223	0.649	0.279	0.649
AttentiveFP	No	4	0.392	0.862	0.390	0.857	0.275	0.901
AttentiveFP	No	4	0.235	0.910	0.541	0.872	0.312	0.798
AttentiveFP	Yes	4	0.257	0.917	0.274	0.826	0.393	0.899
AttentiveFP	Yes	4	0.344	1.047	0.346	1.013	0.161	1.050
AttentiveFP	No	5	0.146	1.137	0.532	1.008	0.221	1.070
AttentiveFP	No	5	0.169	1.178	0.201	1.029	0.359	0.376

AttentiveFP	Yes	5	0.214	1.101	0.398	0.867	0.361	0.824
AttentiveFP	Yes	5	0.243	0.862	0.330	0.806	0.308	0.851
AttentiveFP	No	6	0.318	0.818	0.203	0.972	0.287	0.909
AttentiveFP	No	6	0.079	1.103	0.336	1.027	0.311	1.013
AttentiveFP	Yes	6	0.170	1.008	0.276	0.979	0.227	1.030
AttentiveFP	Yes	6	0.261	1.030	0.091	1.017	0.201	0.992
AttentiveFP	No	7	0.196	0.486	0.220	0.466	0.172	0.412
AttentiveFP	No	7	0.232	0.430	0.296	0.424	0.229	0.414
AttentiveFP	Yes	7	0.261	0.386	0.182	0.420	0.242	0.410
AttentiveFP	Yes	7	0.056	0.457	0.077	0.434	0.041	0.418
TransformerC.	No	1	0.341	0.748	0.334	0.709	0.328	0.709
TransformerC.	No	1	0.323	0.688	0.294	0.706	0.353	0.750
TransformerC.	Yes	1	0.311	0.701	0.371	0.708	0.387	0.713
TransformerC.	Yes	1	0.236	0.681	0.231	0.680	0.224	0.682
TransformerC.	No	2	0.234	0.651	0.197	0.670	0.248	0.703
TransformerC.	No	2	0.217	0.676	0.293	0.688	0.288	0.688
TransformerC.	Yes	2	0.260	0.663	0.287	0.675	0.370	0.686
TransformerC.	Yes	2	0.274	0.647	0.364	0.640	0.304	0.655
TransformerC.	No	3	0.325	0.704	0.298	0.648	0.342	0.638
TransformerC.	No	3	0.161	0.660	0.195	0.671	0.267	0.679
TransformerC.	Yes	3	0.189	0.657	0.303	0.641	0.221	0.653
TransformerC.	Yes	3	0.204	0.645	0.223	0.649	0.279	0.649
TransformerC.	No	4	0.392	0.862	0.390	0.857	0.275	0.901
TransformerC.	No	4	0.235	0.910	0.541	0.872	0.312	0.798
TransformerC.	Yes	4	0.257	0.917	0.274	0.826	0.393	0.899
TransformerC.	Yes	4	0.344	1.047	0.346	1.013	0.161	1.050
TransformerC.	No	5	0.146	1.137	0.532	1.008	0.221	1.070
TransformerC.	No	5	0.169	1.178	0.201	1.029	0.359	0.376
TransformerC.	Yes	5	0.214	1.101	0.398	0.867	0.361	0.824
TransformerC.	Yes	5	0.243	0.862	0.330	0.806	0.308	0.851
TransformerC.	No	6	0.318	0.818	0.203	0.972	0.287	0.909
TransformerC.	No	6	0.079	1.103	0.336	1.027	0.311	1.013
TransformerC.	Yes	6	0.170	1.008	0.276	0.979	0.227	1.030
TransformerC.	Yes	6	0.261	1.030	0.091	1.017	0.201	0.992
TransformerC.	No	7	0.196	0.486	0.220	0.466	0.172	0.412
TransformerC.	No	7	0.232	0.430	0.296	0.424	0.229	0.414
TransformerC.	Yes	7	0.261	0.386	0.182	0.420	0.242	0.410
TransformerC.	Yes	7	0.056	0.457	0.077	0.434	0.041	0.418
Chemprop	-	2	0.130	0.121	0.135	0.634	0.622	0.605
Chemprop	-	2	0.105	0.121	0.142	0.625	0.626	0.621
Chemprop	-	3	0.133	0.129	0.136	0.832	0.831	0.824
Chemprop	-	3	0.120	0.112	0.109	0.838	0.814	0.817

Chemprop	-	4	0.128	0.112	0.150	0.760	0.753	0.750
Chemprop	-	4	0.103	0.108	0.125	0.773	0.768	0.700
Chemprop	-	5	0.122	0.159	0.230	0.428	0.500	0.424
Chemprop	-	5	0.134	0.155	0.244	0.475	0.418	0.418
Chemprop	-	6	0.142	0.120	0.144	0.358	0.374	0.358
Chemprop	-	6	0.207	0.110	0.127	0.403	0.344	0.368
Chemprop	-	7	0.171	0.134	0.153	0.501	0.515	0.486
Chemprop	-	7	0.170	0.139	0.184	0.505	0.514	0.493
MolGpKa	-	2	0.367	0.377	0.358	0.679	0.693	0.704
MolGpKa	-	2	0.487	0.365	0.361	0.670	0.674	0.645
MolGpKa	-	3	0.404	0.365	0.382	0.851	0.948	0.865
MolGpKa	-	3	0.345	0.351	0.331	0.940	0.785	0.913
MolGpKa	-	4	0.451	0.346	0.364	0.866	0.858	0.956
MolGpKa	-	4	0.340	0.339	0.338	0.908	0.846	0.886
MolGpKa	-	5	0.434	0.349	0.375	0.529	0.532	0.662
MolGpKa	-	5	0.327	0.385	0.390	0.534	0.596	0.632
MolGpKa	-	6	0.334	0.356	0.322	0.501	0.532	0.489
MolGpKa	-	6	0.381	0.337	0.313	0.602	0.491	0.500
MolGpKa	-	7	0.355	0.318	0.363	0.589	0.611	0.620
MolGpKa	-	7	0.345	0.439	0.498	0.584	0.660	0.670

Supplementary Table 4. Numerical results for our pKaLearn models (4 graph layers, mask size of 4) in Table 2 and Table 3.

Model	Set	metrics	run#1	run#2	run#3	run#4	run#5	run#6
GATv2Conv	Novartis	MAE	0.512	0.668	0.691	0.272	0.631	0.699
		RMSE	0.859	0.837	0.898	0.860	0.906	0.932
GATv2Conv	Baltruschat	MAE	0.261	0.331	0.365	0.277	0.312	0.319
		RMSE	0.519	0.521	0.579	0.499	0.562	0.484
GATv2Conv	EuroSAMPL	MAE	0.652	0.648	0.654	0.648	0.671	0.695
		RMSE	0.879	0.819	0.84	0.875	0.83	0.938
GATv2Conv	SAMPL8	MAE	1.019	0.51	0.572	0.619	0.768	0.717
		RMSE	1.353	0.654	0.739	0.948	1.226	1.035
Chemprop	EuroSAMPL	MAE	0.767	0.868	0.694	0.762	0.738	0.738
		RMSE	1.082	1.225	1.048	1.147	1.038	1.127
Chemprop	SAMPL8	MAE	0.624	0.666	0.562	0.662	0.768	0.754
		RMSE	0.826	0.875	0.806	0.834	0.952	0.945
MolGpKa	EuroSAMPL	MAE	0.767	0.868	0.694	0.762	0.738	0.738
		RMSE	0.992	0.914	0.725	0.974	0.916	0.869
MolGpKa	SAMPL8	MAE	0.624	0.666	0.562	0.662	0.768	0.754
		RMSE	1.105	0.876	0.835	1.094	1.217	0.953

Supplementary Table 5. Performance (MAE) of our pKaLearn models (models 4-4, trained with 50 randomized graphs or a single non-randomized graph per molecule) on randomized SMILES representations vs. Chemprop and MolGpKa. The error is the standard deviation over the 6 runs (full data in Table S6).

Entry	Training set	Testing set	pKaLearn models (trained with randomized graphs)		Our models (trained with single graphs)		Chemprop	MolGpKa
			w/out IC	w/ IC	w/out IC	w/ IC	w/out IC	w/out IC
1	Training set	Testing set	0.63 ± 0.02	0.62 ± 0.02	0.59 ± 0.02	0.59 ± 0.01	-	-
2	Training set no carbon[a]	Testing set no carbon[a]	0.59 ± 0.02	0.59 ± 0.01	0.56 ± 0.01	0.59 ± 0.01	0.62 ± 0.01	0.68 ± 0.02
3	FS[b] – (Novartis set[c] and analogues)	Novartis set[c]	0.72 ± 0.04	0.75 ± 0.03	0.67 ± 0.02	0.71 ± 0.04	0.83 ± 0.01	0.88 ± 0.06
4	FS[b] – Novartis set[c]	Novartis set[c]	0.66 ± 0.02	0.72 ± 0.02	0.66 ± 0.03	0.71 ± 0.01	0.75 ± 0.03	0.86 ± 0.04
5	FS[b] – (Baltruschat set[c] and analogues)	Baltruschat set[c]	0.40 ± 0.03	0.40 ± 0.03	0.45 ± 0.02	0.46 ± 0.02	0.44 ± 0.03	0.56 ± 0.06
6	FS[b] – Baltruschat set[c]	Baltruschat set[c]	0.32 ± 0.01	0.32 ± 0.03	0.38 ± 0.07	0.38 ± 0.06	0.38 ± 0.02	0.50 ± 0.04
7	Random training set[d]	Random testing set[d]	0.49 ± 0.01	0.50 ± 0.01	0.49 ± 0.02	0.50 ± 0.02	0.50 ± 0.01	0.61 ± 0.04
8	Training set no carbon[a]	EuroSAMPL	0.66 ± 0.02	0.69 ± 0.02	0.64 ± 0.07	0.73 ± 0.06	0.66 ± 0.08	0.81 ± 0.10
9	Training set no carbon[a]	SAMPL8[e]	0.67 ± 0.18	0.62 ± 0.15	0.70 ± 0.08	0.66 ± 0.05	0.75 ± 0.06	0.64 ± 0.06

[a] Training and testing sets used in other investigations above excluding molecules with carbon atoms as ionization centres (IC). [b] FS: our full set excluding molecules with carbon atoms as ionization centres (carbons are not considered as ionization centres by MolGpKa). [c] Novartis: Novartis and Literature dataset as described in [87]. [d] Random training and testing sets used above excluding molecules with carbon atoms as ionization centres. [e] The ionization centres of the SAMPL8 have been assigned using our model inference and our chemical knowledge (Supplementary Figure 10).

Supplementary Table 6. Numerical results (MAE) for our pKaLearn models in Table S5 (ionization center -IC- provided or not, randomized graphs – RG – while training or not, 4 graph layers and a mask size of 4) as well as for Chemprop and MolGpKa when tested on randomized SMILES.

Model	RG	IC	Entry	run#1	run#2	run#3	run#4	run#5	run#6
GATv2Conv	No	No	1	0.605	0.581	0.601	0.604	0.576	0.571
GATv2Conv	No	Yes	1	0.595	0.593	0.578	0.603	0.595	0.573
GATv2Conv	No	No	2	0.552	0.575	0.561	0.556	0.564	0.553
GATv2Conv	No	Yes	2	0.579	0.593	0.585	0.586	0.590	0.571
GATv2Conv	No	No	3	0.668	0.687	0.663	0.638	0.661	0.686
GATv2Conv	No	Yes	3	0.716	0.793	0.696	0.711	0.692	0.755
GATv2Conv	No	No	4	0.690	0.652	0.678	0.638	0.622	0.666
GATv2Conv	No	Yes	4	0.720	0.710	0.732	0.703	0.701	0.719
GATv2Conv	No	No	5	0.470	0.463	0.431	0.410	0.444	0.460
GATv2Conv	No	Yes	5	0.467	0.466	0.422	0.415	0.447	0.465
GATv2Conv	No	No	6	0.302	0.311	0.430	0.348	0.417	0.454
GATv2Conv	No	Yes	6	0.301	0.316	0.425	0.356	0.410	0.457
GATv2Conv	No	No	7	0.467	0.479	0.508	0.501	0.478	0.491
GATv2Conv	No	Yes	7	0.481	0.494	0.517	0.500	0.490	0.519
GATv2Conv	No	No	8	0.302	0.311	0.430	0.348	0.417	0.454
GATv2Conv	No	Yes	8	0.301	0.316	0.425	0.356	0.410	0.457
GATv2Conv	No	No	9	0.467	0.479	0.508	0.501	0.478	0.491
GATv2Conv	No	Yes	9	0.481	0.494	0.517	0.500	0.490	0.519
GATv2Conv	Yes	No	1	0.644	0.648	0.666	0.620	0.610	0.614
GATv2Conv	Yes	Yes	1	0.641	0.623	0.618	0.619	0.598	0.601
GATv2Conv	Yes	No	2	0.562	0.582	0.588	0.591	0.581	0.626
GATv2Conv	Yes	Yes	2	0.584	0.589	0.595	0.579	0.576	0.606
GATv2Conv	Yes	No	3	0.709	0.660	0.670	0.722	0.745	0.749
GATv2Conv	Yes	Yes	3	0.717	0.739	0.711	0.780	0.769	0.792
GATv2Conv	Yes	No	4	0.672	0.653	0.651	0.636	0.686	0.699
GATv2Conv	Yes	Yes	4	0.712	0.720	0.673	0.685	0.721	0.730
GATv2Conv	Yes	No	5	0.452	0.397	0.375	0.396	0.396	0.372
GATv2Conv	Yes	Yes	5	0.451	0.440	0.378	0.395	0.403	0.375
GATv2Conv	Yes	No	6	0.316	0.316	0.328	0.310	0.344	0.320
GATv2Conv	Yes	Yes	6	0.314	0.320	0.331	0.312	0.345	0.321
GATv2Conv	Yes	No	7	0.510	0.490	0.478	0.495	0.498	0.482
GATv2Conv	Yes	Yes	7	0.513	0.504	0.504	0.492	0.500	0.494
GATv2Conv	Yes	No	8	0.316	0.316	0.328	0.310	0.344	0.320
GATv2Conv	Yes	Yes	8	0.314	0.320	0.331	0.312	0.345	0.321
GATv2Conv	Yes	No	9	0.510	0.490	0.478	0.495	0.498	0.482
GATv2Conv	Yes	Yes	9	0.513	0.504	0.504	0.492	0.500	0.494
Chemprop	-	-	2	0.637	0.623	0.608	0.623	0.626	0.622
Chemprop	-	-	3	0.834	0.828	0.821	0.842	0.811	0.824

Chemprop	-	-	4	0.758	0.748	0.746	0.776	0.771	0.691
Chemprop	-	-	5	0.447	0.508	0.440	0.478	0.426	0.423
Chemprop	-	-	6	0.368	0.383	0.398	0.402	0.362	0.384
Chemprop	-	-	7	0.500	0.516	0.484	0.505	0.514	0.488
MolGpKa	-	-	2	0.670	0.674	0.645	0.702	0.683	0.692
MolGpKa	-	-	3	0.945	0.780	0.889	0.863	0.933	0.837
MolGpKa	-	-	4	0.897	0.837	0.856	0.842	0.861	0.941
MolGpKa	-	-	5	0.534	0.596	0.632	0.529	0.532	0.662
MolGpKa	-	-	6	0.602	0.491	0.500	0.501	0.532	0.489
MolGpKa	-	-	7	0.566	0.641	0.655	0.563	0.622	0.608

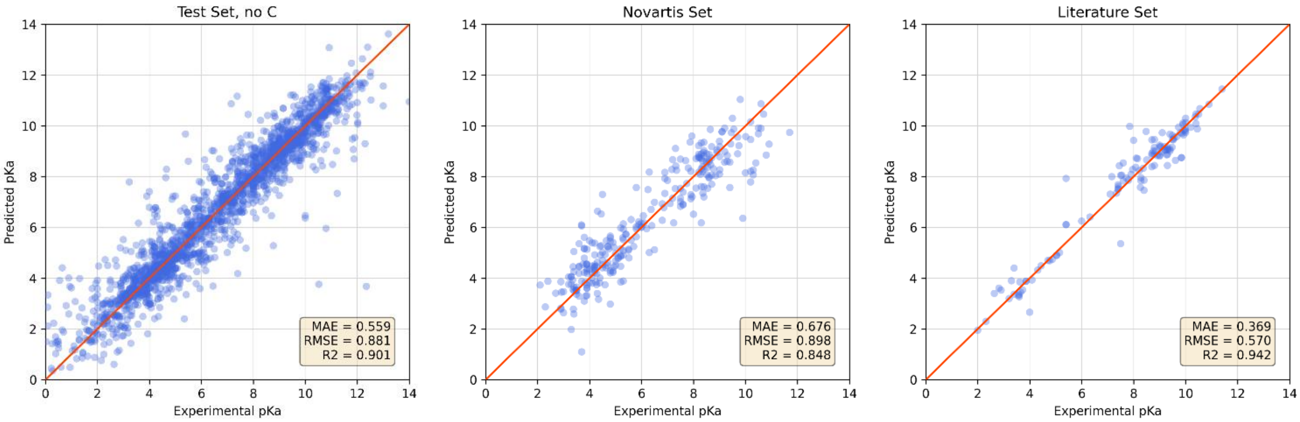

Supplementary Figure 6. pKa predictions of our best pKaLearn model versus the labelled pKa values of our testing set with no carbons (Table 1, Entry 2), on the Novartis Set (Table 1, Entry 3), on the Baltruschat set (Table 1, entry 5). The best-fit linear regression line (perfect prediction) is shown in red.

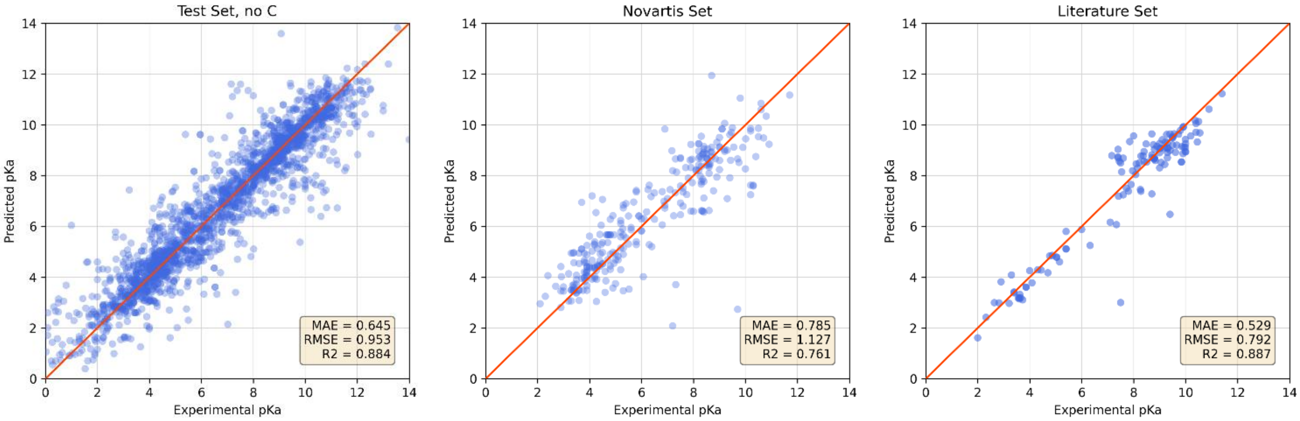

Supplementary Figure 7. pKa predictions of the retrained MolGpKa models versus the labelled pKa values of our testing set with no carbons (Table 1, Entry 2), on the Novartis Set (Table 1, Entry 3) and on the Baltruschat Set (Table 1, Entry 5). The y = x line (perfect prediction) is shown in red.

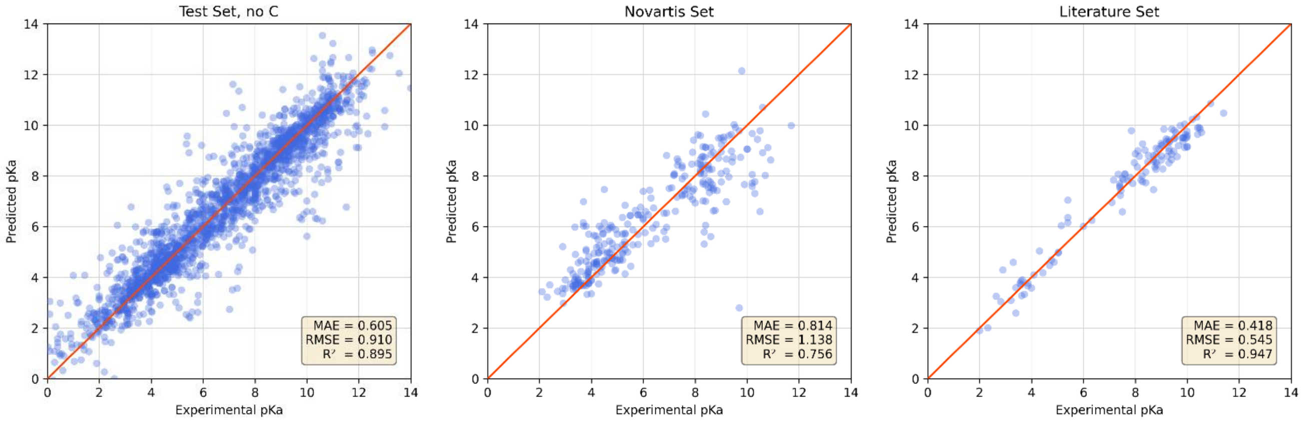

Supplementary Figure 8. pKa predictions of the retrained Chemprop models versus the labelled pKa values of our testing set with no carbons (Table 1, Entry 2), on the Novartis Set (Table 1, Entry 3) and on the Baltruschat Set (Table 1, Entry 5). The y = x line (perfect prediction) is shown in red.

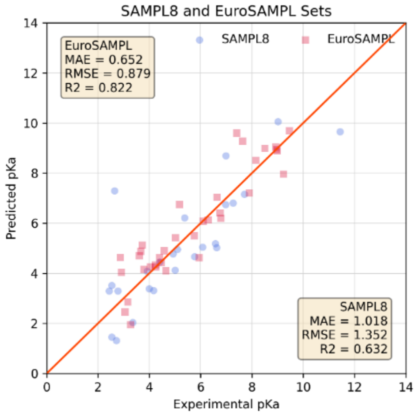

Supplementary Figure 9. pKa predictions of our trained pKaLearn model (Table 1, Entry 2) versus the labelled pKa values of the SAMPL8 set and on the EuroSAMPL data set (Run #1, Table S4). The y = x line (perfect prediction) is shown in red.

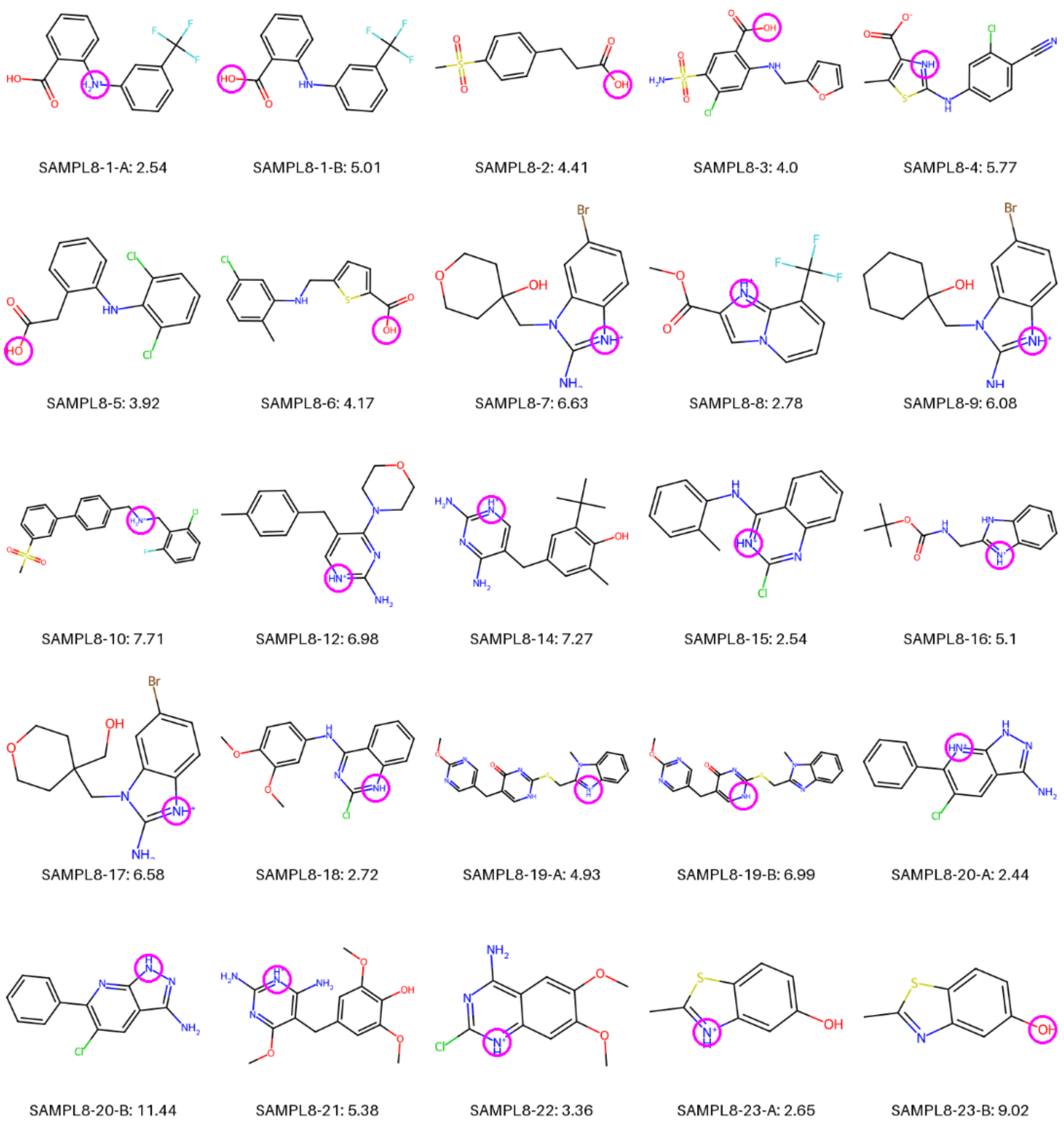

Supplementary Figure 10. SAMPL8 compounds and their experimentally measured pKa values. The ionization center highlighted in pink has been identified by our model and refined using our chemistry expertise.

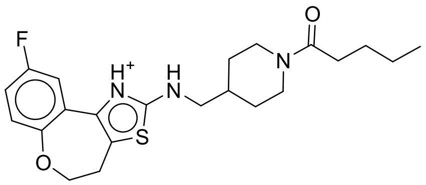

Novartis dataset, pKa = 3.69
model prediction = 6.1

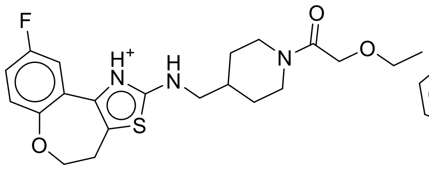

Novartis dataset, pKa = 3.7
model prediction = 6.06

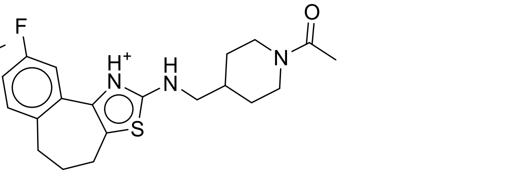

Novartis dataset, pKa = 4.31
model prediction = 6.64

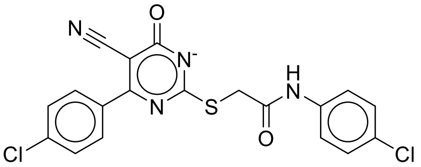

Novartis dataset, pKa = 3.70
model prediction = 1.10

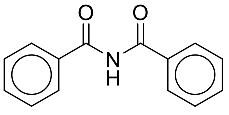

Novartis dataset, pKa = 9.9
model prediction = 6.36

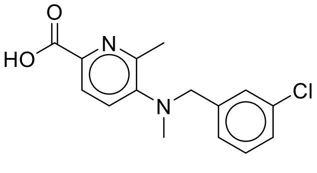

Novartis dataset, pKa = 4.45
model prediction = 6.53

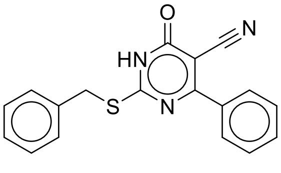

Novartis dataset, pKa = 4.50
model prediction = 7.30

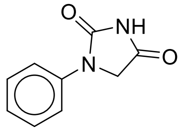

Novartis dataset, pKa = 8.3
model prediction = 6.18

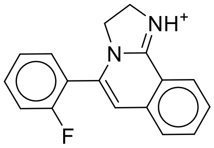

Novartis dataset, pKa = 10.2
model prediction = 7.79

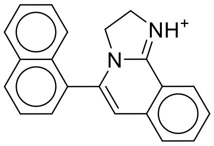

Novartis dataset, pKa = 10.3
model prediction = 8.07

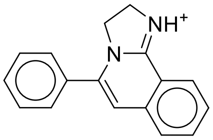

Novartis dataset, pKa = 10.3
model prediction = 8.26

Supplementary Figure 11. Molecules from the Novartis test set and our own test set where our best pKaLearn model prediction has an error larger than 2 units of pKa.

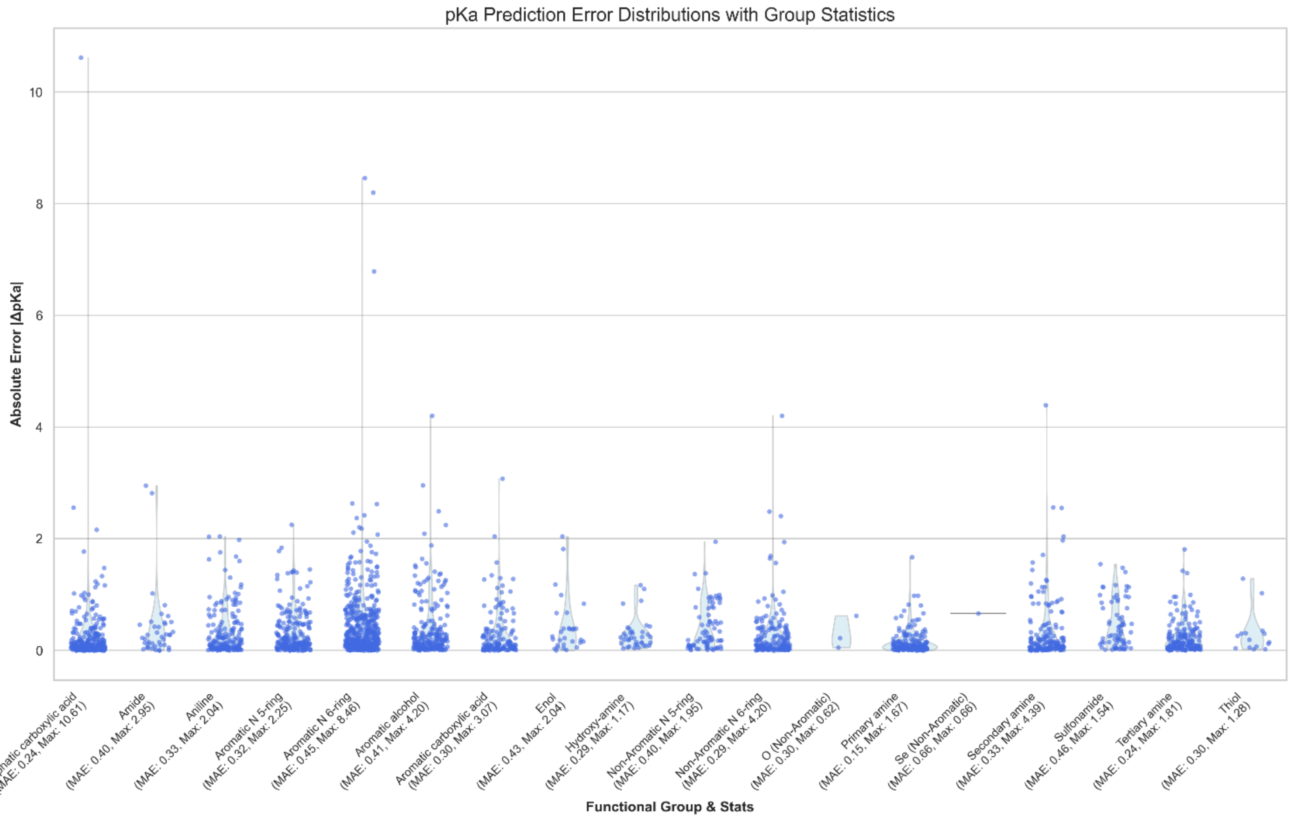

Supplementary Figure 12. Distribution plot of absolute prediction errors (|ΔpKa|) across five different test sets (Testing set no carbon, Novartis Set, Baltruschat Set, EuroSAMPL and SAMPL8) clustered by Functional Group. Each data point represents the mean prediction error for a unique molecule averaged across all testing runs. This represents a set of 2514 unique molecules, not present in any corresponding training runs. The molecules have been classified based on the functional group recognized in the GNN's masking procedure.

S-VI. Code and data availability

Datasets and code can be found here: https://github.com/MoitessierLab/pKaLearn

S-VII. Supplementary References

83. R. C. Johnston, K. Yao, Z. Kaplan, M. Chelliah, K. Leswing, S. Seekins, S. Watts, D. Calkins, J. Chief Elk, S. V. Jerome, M. P. Repasky, J. C. Shelley, *J. Chem. Theor. Comput.* **2023**, *19*, 2380-2388.

84. a) M. Işık, A. S. Rustenburg, A. Rizzi, M. R. Gunner, D. L. Mobley, J. D. Chodera, *J. Comput.-Aided Mol. Des.* **2021**, *35*, 131-166; b) M. Işık, D. Levorse, A. S. Rustenburg, I. E. Ndukwe, H. Wang, X. Wang, M. Reibarkh, G. E. Martin, A. A. Makarov, D. L. Mobley, T. Rhodes, J. D. Chodera, *J. Comput.-Aided Mol. Des.* **2018**, *32*, 1117-1138.

85. P. Hunt, L. Hosseini-Gerami, T. Chrien, J. Plante, D. J. Ponting, M. Segall, *J. Chem. Inf. Model.* **2020**, *60*, 2989-2997.

86. J. L. Suschitzky, P. Sheard, in *Progress in Medicinal chemistry* (Eds.: G. P. Ellis, G. B. West), Elsevier Amsterdam, NY, Oxford, **1984**, pp. 1-62.

87. B. Zdrazil, E. Felix, F. Hunter, E. J. Manners, J. Blackshaw, S. Corbett, M. de Veij, H. Ioannidis, D. M. Lopez, J. F. Mosquera, M. P. Magarinos, N. Bosc, R. Arcila, T. Kizilören, A. Gaulton, A. P. Bento, M. F. Adasme, P. Monecke, G. A. Landrum, Andrew R. Leach, *Nucl. Acids Res.* **2023**.

88 M. Baltruschat, C. P., *F1000Research* **2020**, 113.
89. M. Işık, A. S. Rustenburg, A. Rizzi, M. R. Gunner, D. L. Mobley, J. D. Chodera, *J. Comput. Aided Mol. Des.* **2021**, *35*, 131-166.
90. T. D. Bergazin, N. Tielker, Y. Zhang, J. Mao, M. R. Gunner, K. Francisco, C. Ballatore, S. M. Kast, D. L. Mobley, *J. Comput. Aided Mol. Des.* **2021**, *35*, 771-802
91. B. Weiser, J. Genzling, M. Burai-Patrascu, O. Rostaing, N. Moitessier, *Dig. Discov.* **2023**, *2*, 1841-1849.
92. T. N. Kipf, M. Welling, *arXiv preprint:1609.02907* **2016**.
93. P. Velickovic, G. Cucurull, A. Casanova, A. Romero, P. Lio, Y. Bengio, *Stat* **2017**, *1050*, 20.
94. A. Vaswani, N. Shazeer, N. Parmar, J. Uszkoreit, L. Jones, A. N. Gomez, Ł. Kaiser, I. Polosukhin, *31st Conference on Neural Information Processing Systems (NIPS 2017)*, Long Beach, CA, USA. **2017**.
95. S. Brody, U. Alon, E. Yahav, *arXiv preprint:2105.14491* **2021**.
96. J. Bergstra, D. Yamins, D. Cox, in *Proceedings of the 30th International Conference on Machine Learning, Vol. 28* (Ed.: S. a. Dasgupta, David), PMLR, Atlanta, Georgia, USA, **2013**, pp. 115--123.
